# Comparative progress on the mechanisms of airway mucosal injury induced by different pathogens: SARS-CoV-2, influenza A virus, and Mycoplasma pneumoniae

**DOI:** 10.3389/fcimb.2026.1777403

**Published:** 2026-05-13

**Authors:** Yunlong Jia, Ying Wang

**Affiliations:** 1 Shandong First Medical University, Jinan, China; 2 Respiratory Department No. 960 Hospital, The PLA, Jinan, China

**Keywords:** airway mucosa, comparative mechanisms, epithelial barrier, immunopathology, influenza A virus, Mycoplasma pneumoniae, SARS-CoV-2

## Abstract

Respiratory infectious diseases remain a major global public health challenge. Severe Acute Respiratory Syndrome Coronavirus 2 (SARS-CoV-2), Influenza A Virus (IAV), and Mycoplasma pneumoniae (MP), as three representative respiratory pathogens, all clinically cause airway epithelial shedding, ciliary dysfunction, and Acute Respiratory Distress Syndrome (ARDS). Notably, they are the common triggers of acute respiratory infections characterized by persistent and severe cough, a clinical hallmark rooted in the structural disintegration of the airway mucosal barrier. However, the molecular mechanisms by which they compromise the airway mucosal barrier exhibit significant heterogeneity. Currently, there is a paucity of systematic reviews offering a comparative analysis between these viral and atypical bacterial pathogens. This review comprehensively examines the pathogenic mechanisms of these three agents across four dimensions: receptor recognition, direct cytotoxicity, immunopathology, and abnormal tissue repair. Studies indicate that during the invasion phase, SARS-CoV-2 relies on the Angiotensin-converting enzyme 2 (ACE2) receptor and Transmembrane protease, serine 2 (TMPRSS2) -mediated membrane fusion; IAV identifies sialic acid receptors via hemagglutinin, whereas MP utilizes specialized attachment organelles for “gliding” colonization. Regarding cellular injury mechanisms, SARS-CoV-2 primarily hijacks the endoplasmic reticulum (ER) to induce stress responses and promote syncytium formation; IAV predominantly targets mitochondria to trigger apoptosis and cellular necrosis; while MP utilizes hydrogen peroxide and Community-Acquired Respiratory Distress Syndrome (CARDS) toxin to implement oxidative damage and vacuolating toxicity. At the immunopathological level, SARS-CoV-2-induced delayed interferon response and cytokine storm, IAV-triggered excessive formation of neutrophil extracellular traps (NETs), and MP-mediated activation of the NOD-like receptor thermal protein domain associated protein 3 (NLRP3) inflammasome are key drivers exacerbating airway injury. Furthermore, distinct acute injury mechanisms determine differentiated long-term prognoses, such as pulmonary fibrosis, airway hyperresponsiveness, and airway remodeling. In summary, elucidating the commonalities and specificities of these mechanisms has significant clinical guidance value for precisely distinguishing clinical phenotypes, predicting disease progression, and developing host-directed therapies targeting specific injury pathways.

## 1 Introduction

Respiratory infectious diseases have consistently been a major challenge to global public health (Li et al., 2024). From Severe Acute Respiratory Syndrome Coronavirus 2 (SARS-CoV-2) (Bai et al., 2022), which triggered a global pandemic, to the seasonally epidemic Influenza A Virus (IAV) (Zhu Y. et al., 2025), and Mycoplasma pneumoniae (MP), a common pathogen of community-acquired pneumonia in children and adolescents (Gao and Sun, 2024), these pathogens not only cause Acute Respiratory Distress Syndrome (ARDS) but may also induce long-term lung function impairment. As the host’s first line of physical and immune defense, the airway mucosa serves as the portal for pathogen invasion and the primary battlefield for host-pathogen interactions (Invernizzi et al., 2020).

Despite a substantial volume of research regarding the pathogenic mechanisms of individual pathogens, there is a lack of systematic reviews providing a horizontal comparison of these three representative pathogens (viruses and atypical bacteria). Research has found that SARS-CoV-2, IAV, and the atypical bacterium MP—three pathogens with vastly different biological characteristics—all tend to induce severe coughing (Nypaver et al., 2021; Nguyen et al., 2023; Gavaud et al., 2025). Coughing is a protective reflex; in respiratory tract infections, it is often caused by a significant increase in sensitivity resulting from the exposure of subepithelial sensory nerve endings following damage to the mucous membrane (Naqvi et al., 2023). Therefore, conducting joint research on these pathogens can help identify common mechanisms of airway damage that transcend microbial classification. In fact, although they all lead to similar clinical pathological changes, such as airway epithelial cell shedding, ciliary dysfunction, and inflammatory infiltration during infection, the underlying molecular mechanisms exhibit significant differences. For instance, SARS-CoV-2 utilizes Angiotensin-Converting Enzyme 2 (ACE2) as a receptor and hijacks the host metabolic machinery (Djomkam et al., 2020); IAV binds to sialic acid receptors via hemagglutinin (HA) and induces mitochondrial apoptosis (Zhao and Pu, 2022); whereas MP, as an extracellular pathogen, “glides” via specialized attachment organelles and secretes hydrogen peroxide and Community-Acquired Respiratory Distress Syndrome (CARDS) toxin to directly damage the epithelium (Jiang et al., 2021). Furthermore, the heterogeneity of the host innate immune response is a key determinant of disease severity. The immune evasion and delayed IFN response induced by SARS-CoV-2 often lead to a fatal “cytokine storm” in later stages (Miorin et al., 2020), whereas in IAV infection, the excessive infiltration of neutrophils and the formation of their extracellular traps constitute significant drivers of acute lung injury (Hartshorn, 2020). Elucidating the distinct nuances of these mechanisms is pivotal for differentiating clinical phenotypes, predicting disease progression, and formulating specific intervention strategies.

This article will deeply compare the molecular mechanisms of airway mucosal injury caused by SARS-CoV-2, IAV, and MP from four dimensions: receptor recognition and invasion pathways, direct cytotoxicity mechanisms, innate immunopathology, and abnormal tissue repair, and will discuss their implications for clinical treatment and prognosis management.

## 2 Overview of the structure and function of the airway mucosal barrier

**Figure 1:**
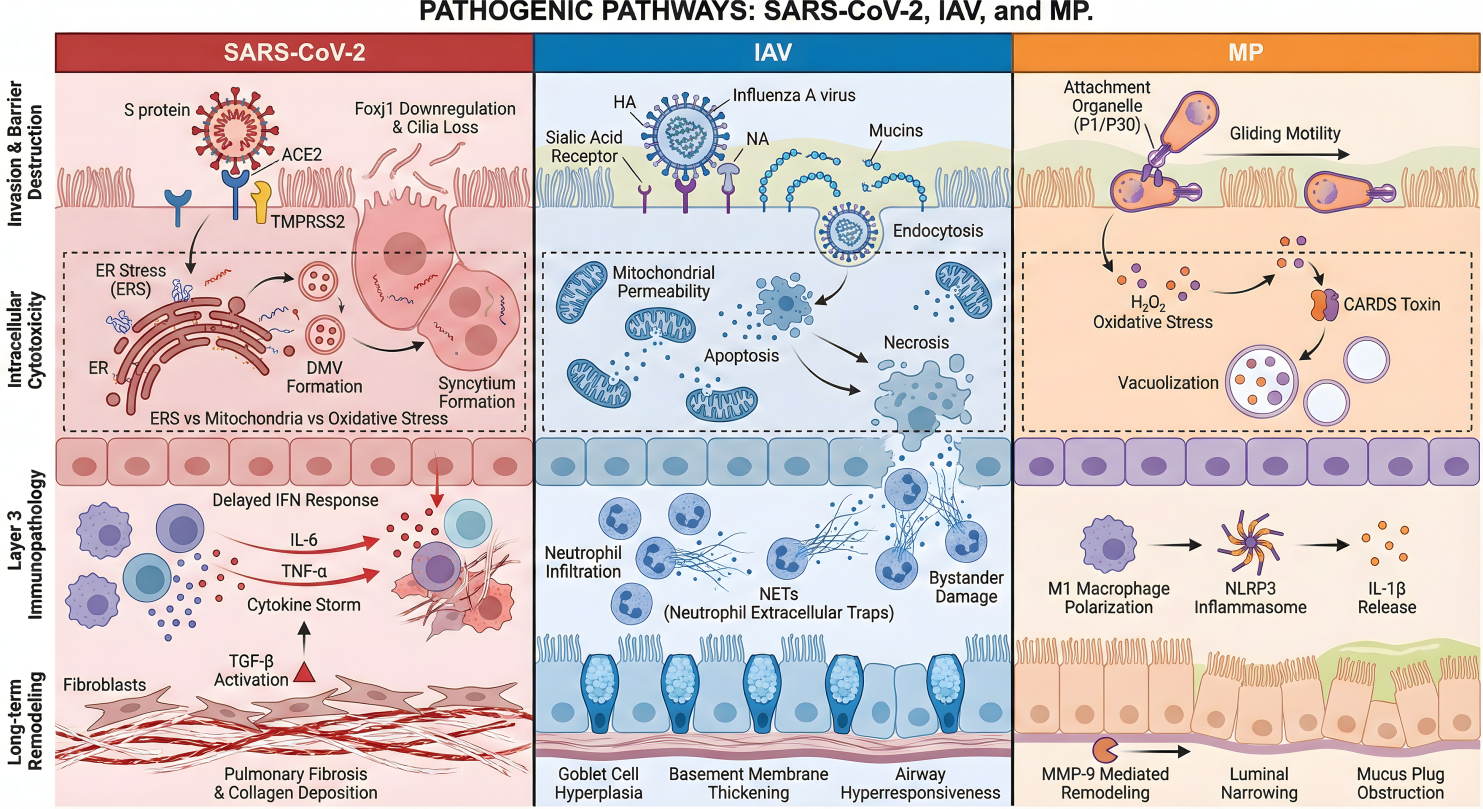
Comparative molecular mechanisms of airway mucosal injury induced by SARS-CoV-2, IAV, and MP. This schematic illustrates the divergent pathogenic pathways across the airway mucosal barrier. **(A)** Entry Phase: SARS-CoV-2 targets ACE2-expressing multiciliated cells; IAV binds to sialic acid receptors; while MP utilizes a specialized attachment organelle for gliding colonization. **(B)** Cytotoxicity Phase: SARS-CoV-2 hijacks the ER to induce ERS and syncytia formation; IAV triggers mitochondrial dysfunction leading to apoptosis; MP produces hydrogen peroxide and CARDS toxin to implement oxidative damage and vacuolization. **(C)** Immunopathology: Key drivers include the delayed IFN-mediated cytokine storm (SARS-CoV-2), excessive neutrophil-derived NETs (IAV), and NLRP3 inflammasome activation (MP). **(D)** Chronic Consequences: Distinct mechanisms lead to pulmonary fibrosis (SARS-CoV-2), airway hyperresponsiveness (IAV), and MMP-9-mediated airway remodeling (MP).

The airway mucosal barrier is the first line of defense maintaining the immune homeostasis of the respiratory system, composed of a precisely coordinated triple defense system: physical, chemical, and immune barriers. The physical barrier is the foundation of defense, primarily consisting of the pseudostratified airway epithelium and its intercellular junctional complexes (Invernizzi et al., 2020). Among these, multiciliated cells and goblet cells operate synergistically to form the “Mucociliary Clearance (MCC)” system via directional beating and mucus secretion, effectively clearing invading particles and pathogens; basal cells serve as a stem cell reserve responsible for epithelial regeneration and repair following injury (Hewitt and Lloyd, 2021; Sheng and Hasnain, 2022). The tight mechanical barrier between epithelial cells relies on structures such as tight junctions and adherens junctions, strictly regulating paracellular permeability and preventing pathogen translocation across the epithelium. The chemical barrier constitutes a sterile interface, primarily mediated by bioactive molecules secreted by the epithelium and glands. The viscoelastic gel layer constructed by mucins is not only the physical carrier for MCC but also exerts a barrier function by trapping pathogens. Additionally, broad-spectrum antimicrobial molecules such as defensins, lysozyme, and lactoferrin not only directly destroy pathogen structures but also exert immunomodulatory functions (Frey et al., 2020). The immune barrier constructs a bridge between innate and adaptive immunity (Marchi et al., 2024). Alveolar macrophages and dendritic cells execute pathogen phagocytosis and antigen presentation functions, respectively, initiating cascading immune responses (Ganjian et al., 2020). At the level of adaptive immunity, secretory IgA (sIgA) produced by plasma cells blocks pathogen adhesion to the epithelium via immune exclusion mechanisms (Hewitt and Lloyd, 2021). Meanwhile, the commensal microbiome colonizing the respiratory tract further consolidates mucosal defense efficacy through niche competition and metabolic regulation (Li et al., 2024). There are significant differences among the three pathogens in terms of the invasion portals, receptor specificity, and the early mechanisms of destruction of the airway physical barrier. For details, please refer to **Table 1**.The integrated molecular pathways of invasion, cellular injury, and immunopathology for the three pathogens are schematically summarized in **Figure 1**.

**Table 1:** Comparison of invasion, receptor recognition, and early airway barrier destruction mechanisms among three pathogens.

Pathogen	SARS-CoV-2	IAV	MP
Pathogen Classification	Enveloped RNA Virus	Enveloped RNA Virus	Cell-wall-less Bacteria
Primary Receptor	ACE2	Sialic acid receptors	Sialylated glycoproteins and sulfated glycolipids
Key Ligand/Adhesin	S protein	HA	Attachment Organelle
Entry/Colonization Mode	Membrane fusion or Endocytosis	Endocytosis	Extracellular adhesion and Gliding motility
Primary Target Cells	ATII cells, nasal goblet cells, and multiciliated cells	Airway epithelial cells and ATII cells	Bronchial ciliated epithelium
Mucus Penetration Mechanism	Passive diffusion or high-affinity binding with ACE2	NA cleaves sialic acid in mucins	Gliding motility and high-efficiency adhesion capability
Direct Ciliary Injury	Downregulation of Foxj1 expression, causing cilia shedding and morphological abnormalities	Ciliary swelling, fusion, and massive shedding	Ciliary stasis, structural disorder, and shedding

## 3 Comparison of invasion (colonization) portals and molecular mechanisms of different pathogens

### 3.1 Receptor recognition and molecular basis of adhesion

The initiation of infection for all three pathogens begins with the specific binding of surface proteins on the pathogen to receptors on host airway epithelial cells; however, their molecular targets and binding mechanisms differ significantly. For Severe Acute Respiratory SARS-CoV-2, the initial step of invading host cells is mediated by the Spike (S) protein, which specifically binds to the ACE2 receptor on the cell surface, thereby promoting virus attachment (Djomkam et al., 2020). The high expression characteristics of ACE2 in type II alveolar epithelial cells, nasal goblet cells, and multiciliated cells determine the respiratory tropism of SARS-CoV-2 and its primary target cell types (Guney and Akar, 2021; Muus et al., 2021). Following S protein binding to ACE2, host transmembrane serine proteases (such as Transmembrane protease, serine 2 (TMPRSS2)) cleave the S protein at the S1/S2 and S2’ sites, triggering major conformational changes in the S protein. This process is a prerequisite for viral fusion, and the utilization efficiency of the ACE2 receptor directly impacts the virus’s transmissibility (Djomkam et al., 2020).

In contrast, successful infection by IAV begins with the specific binding of viral particles to host airway epithelial cells. The HA glycoprotein on the surface of the viral envelope acts as the key molecule, binding to sialic acid receptors (primarily α-2,6-linked sialic acids) on the host cell membrane surface. This specific binding preference determines its infectivity towards human respiratory epithelial cells and is the critical step initiating infection (Zhao and Pu, 2022). It is worth noting that different IAV subtypes exhibit distinct tissue tropism and pathogenic characteristics; for example, seasonal H1N1 viruses primarily target the upper respiratory tract by binding to sialic acid receptors with α-2,6 linkages (Zhu et al., 2022; Zou et al., 2025), whereas avian H5N1 strains can more efficiently infect the lower respiratory tract by binding to receptors with α-2,3 linkages (Nelli et al., 2024; Tan et al., 2025), often leading to more severe alveolar necrosis and fatal immunopathological reactions.

Cell adhesion of MP is a complex process involving multiple factors, primarily mediated by the attachment organelle located at one end of the bacterial body (Jiang et al., 2021). This structure is enriched with various adhesion-related proteins, including P1, P30, P40, P90, P116, P65 proteins, and High Molecular Weight (HMW) proteins 1, 2, and 3 (Jiang et al., 2021; Yiwen et al., 2021). Among them, the P1 protein, as the adhesion protein with the largest molecular weight, is localized at the tip of the attachment organelle and primarily functions to mediate the physical adhesion of MP to host cells (Vizarraga et al., 2020). The P30 protein shares sequence homology with the P1 protein in certain domains, and P30 is located at the tip of the adhesin, playing a critical role in signal transduction to the host cell, cell adhesion, and gliding motility (Zuo et al., 2024). The P30 protein not only directly binds sialylated and sulfated oligosaccharide receptors, inducing metabolic and ultrastructural changes in host cells, but the functional exertion of the P1 protein is also highly dependent on the presence of the P30 protein. MP attaches to bronchial ciliated epithelium through these proteins, leading to pathological changes in the metabolism and ultrastructure of infected respiratory cells.

### 3.2 Cell entry, fusion, and colonization pathways

Upon completing initial adhesion, viruses and mycoplasma adopt distinct strategies to breach the physical barrier or enter the cellular interior (Djomkam et al., 2020). For SARS-CoV-2, following conformational changes in the S protein, it mediates the fusion of the viral envelope with the host cell membrane, completing the internalization of the viral genome. In addition to the direct plasma membrane fusion pathway mediated by TMPRSS2, the virus can also enter cells via clathrin-mediated endocytosis and be activated within endosomes by cathepsins (such as Cathepsin L) (Takeda, 2022). Research has found that TMPRSS2 activity is one of the key factors influencing the transmission of SARS-CoV-2 (Hoffmann et al., 2020). Upon entering the cell, its genome encodes a large polyprotein that is cleaved by Non-structural Protein (NSP) 3 (papain-like protease) and NSP5 (3C-like protease) to generate 16 NSPs (NSP1-NSP16) (Gordon et al., 2020; Lei et al., 2020; Ma C. et al., 2020; Ma X. et al., 2020; Lee et al., 2021). The core function of these proteins is to inhibit host antiviral responses and promote viral replication. Specifically, NSP1 plays a key role in the viral life cycle through immune evasion and translation inhibition, with its primary mechanism of action involving interactions with the small subunit (40S) and NXF1 (Low et al., 2022). NSP3 exhibits multiple immune evasion mechanisms that promote viral replication, with its primary mechanism involving the ability of pLpro to target ISG15 modification. Meanwhile, NSP6 protects viral production by blocking endoplasmic reticulum (ER) -induced autophagosome/autolysosome formation (Low et al., 2022).

For IAV, after binding to the receptor, the virus enters the cell primarily via clathrin-mediated endocytosis or micropinocytosis, forming an endosome. The acidic environment within the endosome is the key factor triggering irreversible conformational changes in HA; this change mediates the fusion of the viral envelope with the endosomal membrane, thereby releasing the viral ribonucleoprotein complex into the cytoplasm, which is subsequently transported to the nucleus for viral genome transcription and replication (Benton et al., 2020; Flórido et al., 2021).

Unlike the mechanisms of viral entry into the cell interior, MP, by virtue of its minute size and high-efficiency adhesion capability, can penetrate the airway mucus barrier and firmly attach to the host ciliated epithelium, achieving colonization (Yiwen et al., 2021). Under the synergistic action of various auxiliary adhesion proteins, the P1 protein facilitates MP to firmly attach and conceal itself on the surface of respiratory epithelial cells, thereby effectively resisting clearance by ciliary movement and phagocytosis by phagocytes. Furthermore, the P1 protein participates in the gliding motility of MP on the host cell surface; this characteristic aids in its further colonization, tissue invasion, and potential systemic dissemination (Zuo et al., 2024). Research shows that this unique adhesion is a key virulence factor of MP, which, by evading host MCC and immune surveillance mechanisms, not only promotes persistent microbial colonization but also lays the foundation for subsequent invasion processes (Sun et al., 2025).

### 3.3 Auxiliary factors, variant characteristics, and pathological consequences

Beyond core adhesion and entry mechanisms, each pathogen relies on specific auxiliary proteins or enzymes to enhance invasiveness, and the invasion process itself can trigger specific pathophysiological changes.

During the variation and evolution of SARS-CoV-2, the emergence of variants such as Omicron brought significant changes to invasion mechanisms. During the evolution of SARS-CoV-2, variants such as Delta and Omicron exhibit distinct tissue tropisms and pathological signatures. The Delta variant possesses high fusogenicity and efficiently utilizes the TMPRSS2 protease for membrane fusion (Meng et al., 2022), allowing it to predominantly target the lower respiratory tract, including type II alveolar epithelial cells (Suzuki et al., 2022). This process leads to extensive syncytia formation. In contrast, the Omicron variant shows reduced cleavage efficiency of the Spike protein and a lower recognition affinity for TMPRSS2. Consequently, Omicron relies more on clathrin-mediated endocytosis and exhibits a tropism shifted toward the upper respiratory tract (Hui et al., 2022; Shuai et al., 2022), where TMPRSS2 expression is relatively lower. This shift correlates with significantly reduced syncytia formation and milder cellular cytotoxicity (Strobelt et al., 2023), explaining its attenuated pathogenicity compared to earlier variants. Additionally, ACE2 is not only a functional receptor for the virus; its internalization and degradation following viral binding lead to a local imbalance of the Renin-Angiotensin System (RAS), with elevated Angiotensin II levels. This process may further exacerbate pulmonary inflammatory responses and increase vascular permeability (Antony and Vijayan, 2021; Beyerstedt et al., 2021).

For IAV, upon completion of viral replication, newly generated viral particles are released via budding, a process dependent on another key envelope protein—Neuraminidase (NA) —and its hydrolytic activity (Strohmeier et al., 2022). NA cleaves sialic acid residues on the cell surface and within the mucus layer, allowing progeny viruses to dissociate from infected cells and penetrate the mucus barrier, thereby mediating effective viral diffusion and transmission within the airway (Creytens et al., 2021; Wu and Ellebedy, 2024). The synergistic action of HA and NA constitutes the molecular basis for IAV to breach airway chemical and physical barriers and achieve highly efficient invasion and transmission.

For MP, in addition to P1 and P30, other adhesion proteins play auxiliary roles during the adhesion process and participate in regulating the localization and function of key adhesion proteins. For example, P90 and P40 proteins are crucial for the insertion of P1 protein into the cell membrane and its transport to the attachment organelle; HMW1 has a significant impact on the structural stability of the attachment organelle, the correct localization of related proteins, and MP adhesion and motility; HMW2 promotes the stability of HMW1, HMW3, P65, and P30 proteins; while HMW3 participates in regulating the localization of P1 and P65 proteins and maintaining P65 protein stability. The P1 protein also simultaneously promotes the release of virulence factors such as hydrogen peroxide and CARDS toxin, thereby damaging host cells. Research also demonstrates that the P1 protein plays an important role in the mast cell inflammatory cytokine response induced by MP, activating mast cells and triggering inflammatory injury through direct contact with sialylated residues on the mast cell surface (Hu et al., 2022).

## 4 Comparison of direct injury mechanisms

### 4.1 Cellular metabolic hijacking and nutritional plundering

After entering the host, the pathogen’s primary task is to utilize host resources to maintain its own survival or replication; this process often directly leads to metabolic dysregulation and functional exhaustion of host cells. SARS-CoV-2 extensively hijacks host metabolism to prioritize its own life cycle, with NSPs and ORF (open reading frame) s playing a critical role (Belizário, 2021). NSP3 and NSP4 recruit ER proteins and double-membrane vesicles (DMVs) for assembly, thereby not only establishing a replication platform but also acting as “lipid traps” that consume large amounts of intracellular lipids and energy (Chen et al., 2022). Concurrently, the virus hijacks VPS39 via ORF3a to inhibit autophagy, thereby ensuring efficient viral release while depriving the cell of its ability to recycle materials and maintain homeostasis (Ivanova et al., 2023). This comprehensive resource depletion leads to “metabolic bankruptcy” and functional failure in host cells, driving the structural disintegration of the airway mucosa.

The high-speed replication of IAV within airway epithelial cells causes multiple direct damages to their structure and function. IAV utilizes the host cell’s transcription and translation machinery to synthesize massive amounts of viral proteins and replicate viral RNA. This process severely hijacks cellular nutrient and energy resources, leading to the inhibition of the host cell’s own protein and RNA synthesis, thereby triggering cellular metabolic disorders (Mifsud et al., 2021; Hui et al., 2022).

MP have small genomes and limited biosynthetic capacity, which means they must rely on their hosts for essential resources such as energy, nutrients, and growth factors (Waites and Talkington, 2004). During its infection, the cell membrane of MP interacts with the host cell membrane, aiding its acquisition of various key compounds necessary for growth and proliferation (Zhang et al., 2021; Hu et al., 2022). The binding of MP to host respiratory epithelium and bronchial ciliated epithelium triggers metabolic and structural changes within affected cells, including cytoskeletal rearrangement. Studies indicate that MP can uptake large quantities of precursor substances such as cholesterol, nucleosides, and amino acids from host cells. This “parasitic plundering” behavior consumes vast amounts of the host cell’s essential nutrients and energy, interfering with normal host cell anabolism, ultimately leading to cellular dysfunction and even death (Jiang et al., 2021; Georgakopoulou et al., 2024).

### 4.2 Oxidative stress, ERS, and organelle dysfunction

Pathogen replication or metabolic products place immense pressure on host organelles (such as mitochondria and the ER and induce intense stress responses. One of the core pathological links in SARS-CoV-2 infection is Endoplasmic Reticulum Stress (ERS). The ER, as the key site for viral replication and protein synthesis, is hijacked by the virus to complete its replication cycle. Coronaviruses rely on the ER as a membrane source to assemble DMVs, providing a physical substrate for viral replication complexes and significantly enhancing RNA synthesis efficiency (Tan et al., 2023). SARS-CoV-2 completes viral assembly and particle secretion by remodeling and utilizing the ERS signal network (Sureda et al., 2020; Upadhyay and Gupta, 2022). Viral proteins (especially M, E, and S proteins) are translated directly on the ER membrane, significantly increasing the protein folding load of the ER, breaking the balance of “folding capacity vs. synthesis demand,” and inducing the Unfolded Protein Response (UPR) and persistent ERS (Sicari et al., 2020; Sureda et al., 2020). IRE1, ATF6, and PERK sensors are all activated, and pathway functions are “reprogrammed” by the virus to adapt to its own replication needs, ultimately leading to tissue injury through the regulation of autophagy and apoptosis (Banerjee et al., 2020; Ha et al., 2020).

IAV-induced mitochondrial injury primarily stems from viral RNA intermediates generated during replication and certain viral proteins (such as PB1-F2, NS1). These components can induce mitochondrial dysfunction and increase membrane permeability, thereby initiating mitochondrial apoptotic pathways (Wang et al., 2021).

MP releases hydrogen peroxide (H_2_O_2_) and superoxide radicals (O_2_^−^) by inserting microtubule structures into host cells. These exogenous reactive oxygen species act together with endogenously produced toxic oxygen molecules within the host cell to induce oxidative stress in respiratory epithelial cells, subsequently leading to biological macromolecule damage, energy metabolism disorders, and activation of inflammatory responses (Sun et al., 2025). MP itself lacks key antioxidant enzymes such as superoxide dismutase and catalase, and the superoxide radicals it produces can inhibit the activity of host cell catalase. This not only reduces the decomposition of peroxides but also enhances the host cell’s sensitivity to the toxic effects of oxygen molecules (Hu et al., 2022). The L-α-glycerophosphate oxidase (GlpD) exposed on its surface serves as a key enzyme in glycerol metabolism, responsible for catalyzing the production of hydrogen peroxide; while this process is beneficial for bacterial survival, it causes severe damage to the host (Schumacher et al., 2019; Sun et al., 2025). Additionally, HPrK, as a central regulatory protein of carbon metabolism, can be activated by glycerol and further exacerbate oxidative stress through peroxide generation. The aforementioned oxidative damage ultimately causes a series of pathological changes in respiratory epithelial cells, including cilia loss, vacuolar degeneration, reduced oxygen consumption, and decreased glucose utilization efficiency.

### 4.3 Cytotoxicity and death mediated by toxins and specific proteins

All three pathogens directly trigger host cell pathology and death by secreting toxins, specific proteins, or inducing the formation of special structures. SARS-CoV-2-mediated cell fusion events and their associated consequences (including syncytia formation and cell-to-cell transmission) are important pathogenic mechanisms (Zhang et al., 2022). SARS-CoV-2 induces syncytia formation through the interaction of S proteins on the surface of infected cells with susceptible cells, involving the participation of the TMPRSS2 protease (Ou et al., 2020; Rajah et al., 2022). Syncytia formation can activate Caspase-9 and Caspase-3/7, ultimately inducing Gasdermin E (GSDME)-mediated pyroptosis (Ma et al., 2021). Furthermore, the M protein can promote cell death by inhibiting the activation of the PDK1-AKT pathway or inducing mitochondrial endogenous apoptosis (Ren et al., 2021; Yang et al., 2022). The NSP6 inhibits IFN production by promoting autophagy-mediated Stimulator of IFN response cGAMP interactor 1 (STING1) degradation and triggers ERS pathways to induce cell autophagy (Sun et al., 2022; Jiao et al., 2023). ACE2-mediated breakthrough infection can also induce inflammatory responses and apoptosis in human bronchial epithelial and microvascular endothelial cells by enhancing autophagy (Li et al., 2021).

IAV can promote the transport of viral ribonucleoprotein complexes (RNPs) out of the nucleus by manipulating apoptosis in type II alveolar epithelial cells, releasing progeny viruses in later stages. The RNP core consists of the RNA polymerase complex proteins PB1, PB2, and PA, as well as nucleocapsid proteins that mediate the binding and packaging of the viral genome. During viral replication, three proteins are expressed that are not incorporated into mature viral particles (Rossman and Lamb, 2011). It is worth noting that NS2 plays a key role in mediating the transport of viral RNP out of the cell nucleus during replication. Additionally, intense viral replication pressure can directly lead to cell necrosis; these two modes of cell death jointly cause rapid loss of epithelial cells and alterations in respiratory function (Zhang et al., 2024).

CARDS toxin is a key virulence factor of MP, possessing unique ADP-ribosylation and vacuolating toxin activities (Hu et al., 2022; Georgakopoulou et al., 2024). The CARDS toxin binds to the Surfactant Protein A (SP-A) receptor on the surface of host target cells, is rapidly internalized via clathrin-mediated endocytosis, and undergoes retrograde transport to the ER, a process that promotes its cleavage and activation (Ramasamy et al., 2021). Interestingly, recent studies have shown that SP-A, through its high-affinity binding to mycoplasma membrane lipid ligands, not only inhibits the growth of MP (as evidenced by reduced colony formation, metabolism, and DNA replication) but also plays a crucial role in establishing antibody-independent immunity against this bacterium (Li et al., 2025b). Therefore, the interaction between CARDS toxin and SP-A serves as a key regulatory node in the bidirectional regulation of defense and pathogenesis in MP. The expression levels, functional status, and genetic polymorphisms of SP-A determine the relative dominance of these two pathways; their dynamic balance profoundly influences disease severity and the progression of lung injury, representing an important pathogenic mechanism that has not yet been fully elucidated (Li et al., 2025b). The CARDS toxin possesses dual functions as a secreted cytotoxin and an adhesin; once inside the cell, its ADP-ribosylation activity disrupts critical cellular functions, leading to characteristic cellular vacuolization, nuclear fragmentation, and ultimate death (Xu et al., 2024; Sun et al., 2025). Moreover, the CARDS toxin induces inflammatory cell death and stress-related diseases by triggering cytokine release, activating the NLRP3 inflammasome, and promoting the release of IL-1βand IL-18 (Su et al., 2021; Hu et al., 2022; Xu et al., 2024).

### 4.4 Disintegration of the airway physical barrier: cilia and tight junctions

All three pathogens ultimately lead to severe destruction of the airway’s first physical line of defense, manifesting as loss of ciliary function and increased epithelial permeability. SARS-CoV-2 preferentially attacks nasal ciliated cells, leading to morphological and size abnormalities in the active cilia of infected cells on one hand, and causing partial ciliary functional defects by downregulating the expression of the ciliogenesis regulator Foxj1 on the other, ultimately resulting in rapid loss of ciliated epithelium (Li et al., 2020; Ahn et al., 2021; Robinot et al., 2021; Ziegler et al., 2021; Chen et al., 2022b). Variants such as Omicron and Delta cause significantly higher degrees of damage to tight junctions compared to early strains (Chiu et al., 2022). This dual destruction mechanism of “ciliated cell injury + tight junction dismantling” directly leads to impaired mucus clearance capabilities and weakened epithelial barrier function, providing a suitable environment for further viral replication and dissemination, and increasing the risk of secondary bacterial infections (Fang et al., 2020).

IAV has a specific tropism for ciliated epithelium; infection can cause ciliary swelling, fusion, collapse, and massive shedding (Guo et al., 2025). More importantly, IAV infection can destroy junctional complexes between epithelial cells by downregulating the expression of tight junction proteins (such as Claudin-4, ZO-1) and adherens junction proteins, significantly increasing epithelial barrier permeability (Short et al., 2016; Lemessurier et al., 2020; Vaswani et al., 2023; Onufer et al., 2025). This loss of barrier function not only facilitates viral diffusion but also allows plasma protein exudation and exacerbates the invasion of foreign pathogens and allergens.

The colonization of MP acts directly on cilia, leading to cilia shedding, structural disorder, and decreased beat frequency, causing severe impairment of airway MCC function (Yamamoto et al., 2019). Concurrently, inflammatory stimulation induces hyperplasia and metaplasia of airway epithelial goblet cells, leading to hypersecretion of mucus and the formation of mucus plugs, which obstruct the airway (Chunlian et al., 2025).

## 5 Comparison of indirect injury mechanisms: innate immunopathology and inflammatory cascades

### 5.1 Activation of innate immune recognition receptors and NF-κB signaling pathways

All three pathogens activate the downstream nuclear factor kappa-B (NF-κB) pathway via pattern recognition receptors, serving as a universal “switch” for initiating inflammatory responses, yet differences exist in receptor specificity and downstream regulation. SARS-CoV-2 triggers the activation of the innate immune system by recognizing and binding to specific pathogen-associated molecular patterns in viral products via pathogen recognition receptors (PRRs), thereby transmitting downstream signals for innate immune activation (Savan and Gale, 2023).

For SARS-CoV-2, host innate immune recognition and NF-κB activation constitute “preparatory steps” for inflammasome activation (Kaivola et al., 2021). Viral nucleic acids can be recognized by human TLR8, triggering the activation of the NF-κB transcription factor; meanwhile, the virus-encoded ORF3a protein can enhance NF-κB activation by modifying the NF-κB subunit p105 via TRAF3-dependent ubiquitination (Kaivola et al., 2021). In addition, once SARS-CoV-2 enters a cell, it can also be recognized by the double-stranded RNA receptor TLR3 and the single-stranded RNA (ssRNA) receptor Toll like receptor (TLR) 7 within endosomes. These receptors transmit signals through their respective key signaling adaptors, TRIF and Myeloid differentiation primary response 88 (MyD88), ultimately inducing type I and type III interferons, interferon-sensitive genes, and pro-inflammatory cytokines (Grigoryan and Pulendran, 2020). Research has found that ssRNA fragments in the SARS-CoV-2 genome can act as direct activators of the endosomal TLR7/8 and MyD88 pathways (Salvi et al., 2021). The expression levels of TLR2 and MyD88 in host cells are positively correlated with the severity of COVID-19; this pathway may exacerbate excessive inflammatory responses by synergistically enhancing NF-κB activation or by directly participating in inflammasome activation (Zheng et al., 2021). The TLR4 signaling pathway, activated during the infection phase, also aggravates injury by inducing oxidative stress and regulating the activation of NETs (Danladi and Sabir, 2021). It is worth noting that various non-structural proteins of SARS-CoV-2 enable the virus to effectively evade the host immune system, leading to increased disease severity and accelerated disease progression (Low et al., 2022). For example, ORF8 evades the host immune system by downregulating MHC-I, while NSP8 interacts with MDA5, impairing its K63-linked polyubiquitination and mediating immune evasion (Rashid et al., 2022).

During IAV infection, host PRRs, such as retinoic acid-induced gene I (RIG-I) and TLRs, recognize conserved viral components, thereby activating innate immune signaling and ultimately inducing the production of various cytokines and antiviral molecules (Chen et al., 2018). For the IAV, its innate immune recognition mechanism exhibits significant cell-type specificity. In human respiratory epithelial cells—the forefront of host defense—the constitutively expressed TLR3 receptor is activated during influenza virus infection; while this process initiates the production of pro-inflammatory cytokines to combat infection, excessive signal transduction often becomes a significant driver, inducing airway pathological responses (Iwasaki and Pillai, 2014). Meanwhile, in plasmacytoid dendritic cells recruited to the infection site, TLR7 plays a core detection role (Iwasaki and Pillai, 2014). TLR7 can specifically recognize the ssRNA genome contained within influenza virus particles ingested into endosomes. Notably, this recognition mode possesses unique kinetic characteristics, occurring without the need for viral replication. Subsequently, via the adapter protein MyD88-mediated signaling cascade, TLR7 rapidly activates NF-κB and IFN Regulatory Factor 7; this synchronous multi-pathway activation collectively constitutes the basis of the early inflammatory response induced by IAV (Iwasaki and Pillai, 2014). To successfully establish infection, IAVs have evolved multiple strategies to evade the host immune system. IAVs utilize various proteins to target different host proteins and evade the host innate immune system (Rashid et al., 2023). For example, the IAV hemagglutinin promotes the ubiquitination and degradation of IFNAR, reducing IFNAR levels and thereby inhibiting the expression of IFN-stimulated antiviral proteins. IAV NS1 is the most important IFN antagonist, acting on multiple targets to suppress the host IFN response. Recent studies have revealed that IAV NP interacts with Mitochondrial Antiviral Signaling Protein (MAVS) and TOLLIP on mitochondria, inducing mitophagy, which degrades MAVS and suppresses the type I interferon response, thereby inhibiting innate immunity (Zhang et al., 2023).

MP can interact with Toll-like receptors (primarily TLR2 and TLR4) on the surface of immune cells (such as macrophages) through its lipoproteins and lipid-associated membrane proteins (Luo et al., 2021; Liu et al., 2022). Following recognition, the NF-κB signaling cascade is activated via a MyD88-dependent pathway, promoting bacterial phagocytosis and clearance, but simultaneously triggering intense inflammatory responses and inducing immune cell apoptosis (Chen et al., 2022a; Georgakopoulou et al., 2024). Additionally, its specific protein MPN606 and lipid-associated membrane proteins can induce macrophage polarization toward the M1 phenotype and massive production of pro-inflammatory cytokines by activating NF-κB and MAPK signaling pathways (Zhang et al., 2025). MP employs various strategies to evade the host’s immune response, thereby ensuring the pathogen’s survival. Its survival mechanisms include immune evasion, which may play a significant role in the pathogenic process (Jiang et al., 2021). For example, MP can use Mpn491 to evade neutrophil-mediated killing (Yamamoto et al., 2017). If the immune response to the invading pathogen is inadequate, it will lead to uncontrolled proliferation of the pathogen and cause damage to host tissues.

### 5.2 Heterogeneity of IFN response: hyperreaction vs. immune evasion

Type I Interferon (IFN) is the core of antiviral immunity; IAV and SARS-CoV-2 exhibit significant differences in the temporal phase of IFN induction and viral antagonism strategies. SARS-CoV-2 has evolved multiple strategies to achieve “delayed response” and multiple blockades. The virus disrupts the RIGI-MAVS pathway activity through the N protein binding to RIG-I and M protein interacting with MAVS; indirectly weakens IFN production by releasing mitochondrial DNA to activate the cGAS-STING1 pathway, leading to cell death (Miorin et al., 2020; Fu et al., 2021). The ORF6 protein severs IFN signal transduction by blocking the nuclear transport of IRF3 and STAT. Proteins such as ORF8, NSP16, NSP1, and NSP8/9 further inhibit host innate immunity across the entire chain by affecting antigen presentation (Banerjee et al., 2020; Xia et al., 2020), inhibiting mRNA translation, and protein transport (Miorin et al., 2020; Tan et al., 2023; Gonzalez-Orozco et al., 2024). This immunosuppressive effect allows the virus to replicate uncontrollably in large quantities during the initial stage of infection, laying the groundwork for the subsequent cytokine storm.

Upon IAV infection of AECII, viral RNA is recognized and activates interferon regulatory factor (IRF)3/7 and NF-κB signaling pathways, inducing rapid expression of IFN-α/β and numerous IFN-stimulated genes (Rashid et al., 2023; Zhang et al., 2024). However, excessive or sustained IFN-I signaling may hinder alveolar epithelial repair and exacerbate epithelial barrier injury during critical periods of lung development or repair (Onufer et al., 2025). In late-stage infection, epithelium-derived IFN-I can also induce alveolar macrophages to express TRAIL, promoting alveolar fluid accumulation and pulmonary edema formation (Lemessurier et al., 2020). As a countermeasure, IAV achieves highly efficient immune evasion through its NS1. NS1 can directly bind viral RNA to shield it from RIG-I recognition, interfere with host mRNA processing, and inhibit IKK activity to interfere with the NF-κB signaling pathway, enhancing viral replication capabilities in multiple aspects (Cruz and Joseph, 2022; Rashid et al., 2023; Zhang et al., 2024).

### 5.3 Neutrophil infiltration, NETs, and “bystander damage”

Excessive activation of neutrophils and the formation of NETs are common pathways by which all three pathogens cause physical destruction of airway tissue. For SARS-CoV-2, the “cytokine storm” recruits neutrophils to aggregate in lung tissue, inducing NET formation and maintaining an inflammatory state. Elevated NET concentrations directly damage lung parenchymal cells and disrupt the homeostasis of the pulmonary microenvironment, serving as a significant cause of poor prognosis and multi-organ injury in patients (Danladi and Sabir, 2021).

Following IAV infection, activated neutrophils release massive amounts of reactive oxygen species, neutrophil elastase, and myeloperoxidase (Hartshorn, 2020; Zhang et al., 2020). When this process becomes uncontrolled, it leads to “bystander damage” to surrounding healthy airway mucosal epithelial cells and endothelial cells, exacerbating tissue edema and epithelial shedding. Neutrophil-mediated uncontrolled inflammatory response is a significant mechanism leading to acute lung injury, ARDS, and high mortality in elderly patients (Kalil and Thomas, 2019; Speaks et al., 2024).

MP can stimulate neutrophils to produce cytokines such as IL-1β and TNF-α, while the CARDS toxin can induce NET formation (Wang et al., 2024). Mediators such as MMP-9, MPO, and NE released by neutrophils, while clearing pathogens, also indiscriminately attack surrounding healthy tissues.

### 5.4 Activation of NLRP3 inflammasome and pyroptosis

NLRP3 inflammasome-mediated pyroptosis is a key mechanism leading to severe inflammation and tissue injury; all three pathogens trigger this pathway via specific molecules. SARS-CoV-2-derived ssRNA sequences activate the NLRP3 inflammasome in human macrophages via a non-canonical inflammasome pathway: SARS-CoV-2 RNA induces TLR8-mediated IL-1β production without thermal attenuation, a process that depends on potassium efflux and NLRP3 (Campbell et al., 2021). These results suggest that both TLR8 and NLRP3 play important roles in defending against SARS-CoV-2 infection. The ORF3a protein, encoded by both SARS-CoV-1 and SARS-CoV-2, is a viroporin that functions as a potassium channel. It is involved in viral particle assembly and membrane budding processes in these viruses. Both SARS-CoV-1 and SARS-CoV-2 ORF3a proteins are believed to be involved in NLRP3 inflammasome activation (Siu et al., 2019; Xu et al., 2022). Specifically, ORF3a interacts with TRAF3 to ubiquitinate Apoptosis-associated speck-like protein containing a CARD (ASC), thereby enhancing NLRP3 inflammasome activation (Kaivola et al., 2021). Recent evidence suggests that the SARS-CoV-2 N protein promotes NLRP3 inflammasome activation and subsequent excessive inflammation. The N protein interacts directly with the NLRP3 protein. This interaction promotes the binding of NLRP3 to ASC and facilitates the assembly of the inflammasome complex, leading to the formation of ASC oligomers (Pan et al., 2021). Furthermore, SARS-CoV-2-secreted N-protein dimers autoactivate MASP-2, the primary enzyme activator of the lectin pathway. Activation of MASP-2 leads to the production of C3/C5 convertase, which subsequently forms the MAC. The MAC complex increases cytoplasmic calcium concentrations, triggering NLRP3 inflammasome activation, followed by the release of IL-1β and GSDMD-mediated delayed release (Kaivola et al., 2021). Studies have found that the SARS-CoV-2 ORF8b protein can interact with the NLRP3 leucine repeat domain, promoting the activation of the NLRP3 inflammasome (Kaivola et al., 2021). The activated inflammasome induces pyroptosis via GSDMD, disrupting cell integrity and releasing pro-inflammatory mediators, which is a critical mechanism in the pathogenesis of severe COVID-19 (Islamuddin et al., 2022).

The NLRP3 inflammasome is a major antiviral host defense mechanism during influenza virus infection. We have previously demonstrated that IAV infection triggers NLRP3 inflammasome assembly, activating caspase-1, which subsequently cleaves GSDMD to produce an N-terminal fragment. This fragment forms a pore in the cell membrane, mediating pyroptosis (Speaks et al., 2024; Zhang et al., 2024). This process not only leads to the lysis of airway epithelial cells but also releases large amounts of IL-1β and IL-18, thereby exacerbating the inflammatory cascade[Hartshorn, 2020; Gonzalez-Orozco et al., 2024]. We have now discovered that multiple IAV proteins can initiate the activation of the NLRP3 inflammasome (Pandey and Zhou, 2022). For example, the PB1-F2 protein activates the NLRP3 inflammasome and induces IL-1β secretion, thereby promoting the pathogenic mechanism of IAV. Several other IAV proteins have also been found to directly or indirectly participate in the activation and regulation of inflammasome activity (Pandey and Zhou, 2022). For example, the PB1-F2 protein activates NLRP3 through multiple mechanisms: the aggregated form of the C-terminal region of PB1-F2 has been shown to activate NLRP3 via mitochondrial action; PB1-F2 translocates to mitochondria, promoting the production of mitochondrial ROS and the release of mitochondrial DNA, which in turn activates NLRP3 (Wang et al., 2021); studies have revealed the critical role of the IFN-induced Z-DNA-binding protein 1 in inflammasome activation during influenza virus infection (Kuriakose et al., 2016). Notably, the IAV M2 protein has also been shown to activate the inflammasome by regulating ion flux or stimulating the release of mitochondrial DNA into the cytoplasm. Specifically, the IAV M2 protein regulates TGN diffusion through its ion channel function; NLRP3 is recruited to the dTGN, where it undergoes depolymerization, a conformational change, and recruits ASC. Consequently, the inflammasome is activated, leading to the production of IL-1β (Pandey and Zhou, 2022). Additionally, both TLR and RIG-I sensors of the influenza virus are involved in regulating inflammasome activation and IL-1β release during infection. The recognition of the virus by these receptors activates various intracellular signaling cascades, including the NF-κB activation pathway (Kuriakose and Kanneganti, 2017). Among these, RIG-I is considered one of the upstream regulators of inflammasome assembly during influenza virus infection, as it can directly form an inflammasome complex with ASC and caspase-1, or indirectly regulate inflammasome assembly by inducing type I IFNs (Kuriakose and Kanneganti, 2017).

The NLRP3 inflammasome can promote inflammation following infection with MP (Zhu M. et al., 2025). Studies have shown that the membrane lipoprotein components of MP can activate the NLRP3 inflammasome via the TLR/NF-κB signaling pathway (Luo et al., 2021). Specifically, inflammation induced by MP lipids is mediated by the TLR-4 pathway, which subsequently triggers the activation of the NLRP3 inflammasome and forms a positive feedback loop between autophagy and the NF-κB signaling cascade, ultimately promoting the production of TNF-α and IL-1β in macrophages (Luo et al., 2021). Furthermore, the CARDS TX produced by MP possesses ADP-ribosyltransferase activity, which can directly catalyze the assembly of the inflammasome via the NLRP3 protein (Song et al., 2024). Activation of the NLRP3 inflammasome promotes the conversion of the precursors of interleukins IL-1β and IL-18 into their mature forms and cleaves the pyroptosis executor protein GSDMD, generating an active N-terminal fragment that subsequently perforates the cell membrane, leading to cell death (Yin et al., 2023). The pyroptosis process not only exacerbates the inflammatory response but also releases pathogen antigens previously phagocytosed by macrophages into the extracellular space, thereby perpetuating the infection (Ding et al., 2021).

### 5.5 Macrophage activation and “cytokine storm”

Uncontrolled cytokine release is the common terminal pathway leading to severe injury and ARDS, with macrophage polarization and specific metabolic products playing central roles. SARS-CoV-2 infection induces virus-carrying alveolar cells to release massive amounts of cytokines (such as IL-6, TNF-α, MCP-1, etc.), forming a “cytokine storm (Parasher, 2021).” This phenomenon leads to severe ARDS, which causes pneumonia and respiratory failure and is considered one of the primary causes of COVID-19-related mortality (Abdin et al., 2020). Macrophages play a key role in this (Wang Chau and Sugimura, 2022; Foo et al., 2023), with their secretion of IL-6 and derived IL-1β and TNF promoting TH17 cell responses, exacerbating pulmonary inflammatory infiltration (Park, 2020; Kaivola et al., 2021). IL-6 plays a key role in this process, mediating its effects through two pathways: the cis (classical) and trans signaling pathways. Once activated, both pathways trigger a series of events that lead to the activation of the JAK-STAT and AKT/PI3K pathways (Hunter and Jones, 2015). IL-6 is also involved in a variety of biological functions, including T-cell clonal expansion, B-cell differentiation, acute inflammatory responses, and mitochondrial activity (Hunter and Jones, 2015). Numerous studies have reported that IL-1β is one of the primary mediators of lung injury and inflammation during viral infection. Upon binding to its receptor, IL-1R, IL-1β activates a cascade of signaling events involving the NF-κB transcription factor, c-Jun N-terminal kinase, and P38 MAPK (Abdin et al., 2020).This excessive inflammatory response may be far more destructive to lung tissue than direct viral invasion, ultimately leading to diffuse alveolar damage and ARDS (Mehta et al., 2020; Yao et al., 2020; Parasher, 2021). Additionally, studies have shown that inflammatory macrophages are considered the primary mediators of lung injury during the exudative phase, whereas regulatory macrophages—formerly referred to as “alternatively activated” or “M2” macrophages—are associated with the proliferative and fibrotic phases of ARDS (Wendisch et al., 2021).

The clinical prognosis and pathogenesis of influenza virus infection in human and animal models are poor, and are often associated with elevated levels of pro-inflammatory cytokines and chemokines—a condition also known as cytokine storm (Wei et al., 2022). This condition precedes ARDS and frequently leads to death. In IAV infection, monocytes are recruited in large numbers and become significant sources of cytokines via pathways such as RIG-I/MAVS and TLRs. Studies have shown that the binding of IAV RNA to the host receptor RIG-I initiates the IAV infection response; once activated, RIG-I triggers the expression of IFN and pro-inflammatory genes (Pitré et al., 2025). IAV infection can activate TLR signaling pathways. It has been reported that sustained activation of TLR3 is detrimental in IAV-induced acute pneumonia. Activation of TLR4 regulates IAV entry and tropism through the regulation of MyD88 expression and p38 MAPK activation. IAV infection can induce severe oxidative stress and acute lung injury via the TLR4-TRIF-TRAF6-NF-κB signaling pathway (Dai et al., 2017). Coagulation disorders induced by viral infection can further promote IL-6 and IL-8 release, exacerbating the cytokine storm (Linfield et al., 2021). IL-6 is a key cytokine in the cytokine storm associated with the pathogenesis of IAV infection. It acts in concert with other pro-inflammatory cytokines, such as TNF-α and IL-1β, to trigger an excessive immune response, causing tissue damage and potentially leading to severe consequences, including organ failure and death (Li et al., 2025a). In severe influenza infections, elevated IL-6 levels are associated with increased disease severity, pulmonary inflammation, and poor clinical outcomes (Gu et al., 2021). Therefore, IL-6 dysregulation may lead to respiratory failure and increase the risk of death.

MP stimulates the host to release inflammatory mediators such as IL-8 and TNF-α through its own components. IL-8 is considered a potent neutrophil activator and has been shown to promote inflammation in the lungs and outside the lungs following MP infection (Chmura et al., 2008). TNF-α induces the recruitment of inflammatory cells, leading to local inflammation, airway remodeling, airflow obstruction, emphysema, and impaired lung function (Chen et al., 2022a). In addition, its HapE protein produces hydrogen sulfide, which stimulates phagocytes to release pro-inflammatory factors and upregulate the expression of inflammatory mediators (Waites et al., 2017; Li et al., 2019; Xue et al., 2023; Georgakopoulou et al., 2024). Elevated CRP levels can also promote lung epithelial cell death via the p38 MAPK/mitochondrial apoptosis pathway, aggravating injury (Li et al., 2025). In summary, although all three pathogens trigger robust inflammatory cascades, they employ distinct molecular pathways in inducing cell death and immune evasion, dictating their unique pathological outcomes, as outlined in **Table 2**.

**Table 2:** Differences in mechanisms of cell death, immune evasion, and inflammatory cascades.

Pathogen	SARS-CoV-2	IAV	MP
Dominant Cell Death Type	Pyroptosis/Apoptosis/Syncytial death	Apoptosis/Necrosis/ Pyroptosis	Vacuolization and lysis/Apoptosis
Core Immune Evasion Molecules	NSP1, NSP6, NSP16, ORF6, ORF8, N, M	NS1 Protein	Mpn491, HapE
IFN (IFN) Antagonism	ORF6 blocks STAT1/2 nuclear import, NSP1 inhibits translation, N protein shields RNA recognition	NS1 binds vRNA and inhibits IKK/NF-κB activation	Induces Th1-type inflammation, inhibits catalase to enhance oxidative stress
Inflammasome Activation	ORF3a, ORF8b, and N protein	PB1-F2 protein, M2 ion channel activity, and vRNA	CARDS toxin, Lipoproteins, and ROS accumulation
Neutrophil Interaction (NETs)	NETs are closely related to organ damage	Excessive NETs lead to severe physical injury of the alveolar epithelium	CARDS induce NETs, but Mpn491 degrades NETs to evade killing
Final Pathological Consequences	Diffuse Alveolar Damage, Microthrombosis	Rapid epithelial shedding, Secondary bacterial co-infection	Ciliary stasis, mucous plug obstruction, and airway hyperresponsiveness/fibrosis

## 6 Comparison of long-term sequelae and abnormal tissue repair

### 6.1 Abnormal tissue repair: airway remodeling and pulmonary fibrosis

The abnormal repair mechanisms following infection by the three pathogens manifest as airway remodeling, pulmonary fibrosis, and epithelial metaplasia, respectively. SARS-CoV-2 infection, particularly in recovered severe cases, often presents with pulmonary fibrosis characterized by reticular shadows or traction bronchiectasis (Hatabu et al., 2023). Activation of the TGF-β signaling pathway promotes the differentiation of fibroblasts into myofibroblasts and excessive ECM deposition, destroying alveolar structure, which is a key mechanism for irreversible Long COVID sequelae (Vaz De Paula et al., 2021).

Recovered severe IAV patients may exhibit goblet cell hyperplasia, squamous metaplasia, basement membrane thickening, and airway hyperresponsiveness, which are associated with post-infection chronic cough and acute exacerbation of asthma (Goncheva et al., 2020; Liu et al., 2021).

MP infection can upregulate MMP-9 expression via TLR2/6 and MAPK/NF-κB pathways and promote the release of factors such as TGF-β and TNF-α, inducing epithelial-mesenchymal transition and cell migration, leading to collagen deposition and airway smooth muscle hyperplasia, ultimately resulting in airway remodeling (Qin et al., 2022; Iannuzo et al., 2023; Yang et al., 2025). This is particularly significant in severe or refractory patients and may cause persistent lung function impairment.

### 6.2 Risk of secondary and co-infections

For IAV and theSARS-CoV-2, secondary or concurrent bacterial infections are very common and are often major drivers of disease progression and mortality (Mirzaei et al., 2020). This phenomenon is underpinned by complex host-pathogen mechanisms that disrupt the multilayered defense systems of the lungs. In contrast, while there have been reports of co-infections between MP and viruses (such as SARS-CoV-2) (Karaaslan et al., 2021), the number of such reports is limited and the mechanisms remain unclear; therefore, this topic is addressed only as a side note.

According to laboratory, clinical, and epidemiological studies, patients with viral infections who also have bacterial infections (whether secondary or concurrent) exhibit a significantly higher mortality rate (Metzger and Sun, 2013). During viral transmission, the infection can cause histological and functional damage to the respiratory tract, including alterations in mucus secretion, cell death, hyperplasia, reduced mucociliary clearance, impaired gas exchange, and compromised surfactant secretion (Bakaletz, 2017). These changes weaken the host’s immune response, thereby creating conditions conducive to viral-bacterial co-infection.

During the COVID-19 pandemic, a large number of immunocompromised patients were hospitalized, and some COVID-19 patients were diagnosed with secondary infections; evidence suggests that multidrug-resistant bacteria are among the common pathogens (Rasmussen et al., 2020). Zhou et al. found that 50% of COVID-19 fatalities during the pandemic involved bacterial co-infections (Zhou et al., 2020). SARS-CoV-2 infection may damage cells and lung structures, and the resulting altered environment enhances bacterial adhesion and invasiveness. The study suggests three possible mechanisms: direct interaction between the virus and co-pathogenic microorganisms; therapeutic interventions promoting co-infection; and the hospital environment increasing infection risk during the pandemic (Lubkin et al., 2024).

The most common cases of viral-bacterial co-infection are observed in individuals infected with influenza viruses (Mirzaei et al., 2020). The sialidase activity of the influenza virus NA can alter carbohydrate groups on the surface of host epithelial cells, creating conditions for bacterial attachment. This enzyme also increases the probability of bacterial attachment to host cells by stimulating TGF-β, which upregulates integrins and fibronectin (Li et al., 2015). Additionally, influenza virus-induced IFNs lead to reduced expression of C-C motif chemokine ligand 2, resulting in failed macrophage recruitment and thereby promoting the colonization of Streptococcus pneumoniae in the body (Nakamura et al., 2011). Studies have also found that influenza viruses render the host susceptible to Staphylococcus aureus pneumonia, with both viral and bacterial loads increasing during co-infection (Iverson et al., 2011). One hypothesis suggests that the increased viral load following co-infection stems from accelerated viral shedding from infected host cells, while the elevated bacterial load is associated with impaired alveolar macrophage function (Smith et al., 2013).

### 6.3 Mechanisms underlying chronic symptoms and post-acute sequelae

In addition to acute damage to the airway mucosa and lung parenchyma, incomplete tissue repair and persistent immune responses following primary infection with SARS-CoV-2, IAV, or MP also serve as the pathological basis for persistent post-acute symptoms and long-term sequelae, rather than merely residual inflammation. These two intertwined mechanisms drive the transition of infection from an acute state to chronic pathology, ultimately resulting in persistent functional impairment of the respiratory system and even multiple extrapulmonary systems.

Following SARS-CoV-2 infection, 31%–69% of recovered patients experience post-acute sequelae of COVID-19, primarily manifested as persistent fatigue, dyspnea, cognitive impairment, and chronic cough (Carfì et al., 2020; Peluso and Deeks, 2024). At the tissue repair level, abnormal activation of the TGF-β signaling pathway leads to persistent extracellular matrix deposition and dysfunction of microvascular endothelial cells (Vaz De Paula et al., 2021); incomplete repair of damaged ATII cells and impaired clearance of residual syncytia further hinder epithelial regeneration. At the immunological level, the primary infection induces persistent low-grade inflammation, sustained activation of the NLRP3 inflammasome (Campbell et al., 2021; Pan et al., 2021), as well as residual delayed IFN responses and excessive complement activation. These unresolved immune responses trigger thrombotic inflammation, tissue hypoxia, and abnormal neuro-immune interactions, which are key factors leading to extrapulmonary sequelae (Hatabu et al., 2023).

Acute post-infection sequelae following IAV infection are primarily characterized by persistent airway hyperresponsiveness, chronic cough, and transient decline in lung function, with these symptoms being more pronounced in severely ill patients, the elderly, and children. Abnormal tissue repair manifests as irreversible goblet cell hyperplasia, squamous metaplasia, and basement membrane thickening; residual NETs and Type I IFN signaling imbalance hinder epithelial barrier repair (Speaks et al., 2024; Onufer et al., 2025). Persistent local inflammation (e.g., sustained secretion of TNF-α and IL-6) maintains hypersensitivity of the airway sensory nerves, directly leading to prolonged post-infection cough and increasing the risk of acute asthma exacerbations. Unlike SARS-CoV-2, the sequelae of IAV are mostly confined to the respiratory tract and rarely cause severe extrapulmonary damage.

Primary MP infection often triggers refractory chronic cough, airway remodeling, and long-term lung function impairment, particularly in children and adolescents. The core mechanisms involve: incomplete epithelial repair leading to persistent ciliary dysfunction and mucociliary clearance failure; MP-induced sustained activation of the NLRP3 inflammasome (Zhu M. et al., 2025), residual oxidative stress, and overexpression of MMP-9 (Qin et al., 2022), which disrupt the balance between tissue degradation and repair, promoting airway smooth muscle proliferation and collagen deposition. Concurrently, MP’s immune evasion mechanisms (such as Mpn491-mediated NET degradation) can lead to low-level persistent colonization and chronic immune stimulation (Yamamoto et al., 2017), maintaining airway inflammation and hyperresponsiveness, which is closely associated with the development of post-infectious wheezing and asthma.

## 7 Conclusion

Through an in-depth comparison of airway mucosal injury mechanisms caused by SARS-CoV-2, IAV, and MP, we find that although these three pathogens cross the biological boundary between viruses and bacteria, they exhibit a pathological outcome of “different paths, same destination” in destroying host defense barriers. Through a comparative analysis, this study found that damage to the physical barrier of the airway mucosa is a common target for all three conditions. A deeper understanding of these mechanisms not only explains their shared clinical manifestation of severe coughing but also provides a precise theoretical foundation for the development of host-targeted therapies aimed at repairing the airway mucosa and alleviating cough symptoms.

In the invasion phase, the three have evolved highly specific receptor recognition mechanisms: SARS-CoV-2’s high-affinity binding to ACE2 and its dependence on TMPRSS2, IAV’s tropism for specific sialic acid chains, and MP’s complex P1/P30 adhesion complex all precisely target airway epithelial cells. However, in intracellular pathogenic mechanisms, they display unique “arsenals”: SARS-CoV-2 primarily hijacks the ER to trigger stress responses and promote syncytia formation; IAV focuses on disrupting mitochondrial integrity and inducing cell necrosis; whereas MP utilizes its unique metabolic products (such as H_2_O_2_) and CARDS toxin to implement oxidative damage and vacuolating toxicity.

Comparison of immunopathology reveals key differences in progression to severe disease. SARS-CoV-2 inhibits IFN signaling through multiple proteins, leading to uncontrolled early viral replication and a subsequent cytokine storm; in IAV infection, excessive activation of neutrophils and NET formation are core drivers of tissue injury, whereas MP-induced activation of the NLRP3 inflammasome and mixed-type immune responses exacerbate chronic inflammation.

Ultimately, these acute injury mechanisms determine distinct long-term prognoses. SARS-CoV-2 tends to induce pulmonary fibrosis via the TGF-β pathway; MP infection is prone to leading to MMP-9-mediated airway remodeling and stenosis, while IAV more commonly causes epithelial metaplasia and airway hyperresponsiveness.

In summary, future therapeutic strategies should not be limited to anti-pathogen treatments alone but should also focus on host-directed therapies targeting specific injury pathways. For example, ERS inhibitors for SARS-CoV-2, NET degradation agents for IAV, and antioxidant therapies for MP represent highly promising research directions. Concurrently, deeply elucidating these mechanisms has important clinical guidance significance for preventing secondary bacterial infections and improving long-term sequelae such as “Long COVID”.

## Glossary

SARS-CoV-2: Severe Acute Respiratory Syndrome Coronavirus 2
IAV: Influenza A Virus
MP: Mycoplasma pneumoniae
ARDS: Acute Respiratory Distress Syndrome
ACE2: Angiotensin-converting enzyme 2
CARDS: Community-Acquired Respiratory Distress Syndrome
NETs: neutrophil extracellular traps
HA: hemagglutinin
MCC: Mucociliary Clearance
sIgA: secretory IgA
S: Spike
HMW: High Molecular Weight
RAS: Renin-Angiotensin System
NSP: Non-structural Protein
ER: Endoplasmic reticulum
ERS: Endoplasmic Reticulum Stress
DMVs: Double-Membrane Vesicles
GlpD: L-α-glycerophosphate oxidase
GSDME: Gasdermin E
STING1: Stimulator of interferon response cGAMP interactor 1
RNPs: ribonucleoprotein complexes
SP-A: Surfactant Protein A
NLRP3: NOD-like receptor thermal protein domain associated protein 3
NF-κB: nuclear factor kappa-B
TLR: Toll like receptor
IFN: Interferon
IRF: interferon regulatory factor
ORF: open reading frame
ssRNA: single-stranded RNA
RIG-I: Retinoic acid-induced gene I
PRRs: pathogen recognition receptors
MAVS: Mitochondrial Antiviral Signaling Protein
MAPK: mitogen-activated protein kinase
NA: Neuraminidase
TMPRSS2: Transmembrane protease, serine 2
ASC: Apoptosis-associated speck-like protein containing a CARD
MyD88: Myeloid differentiation primary response 88.

## References

[B1] Abdin S. M. Elgendy S. M. Alyammahi S. K. Alhamad D. W. Omar H. A . (2020). Tackling the cytokine storm in COVID-19, challenges and hopes. Life Sci. 257, 118054. doi: 10.1016/j.lfs.2020.118054. PMID: 32663575 PMC7832727

[B2] Ahn J. H. Kim J. Hong S. P. Choi S. Y. Yang M. J. Ju Y. S. . (2021). Nasal ciliated cells are primary targets for SARS-CoV-2 replication in the early stage of COVID-19. J. Clin. Invest. 131. doi: 10.1172/jci148517. PMID: 34003804 PMC8245175

[B3] Antony P. Vijayan R. (2021). Role of SARS-coV-2 and ACE2 variations in COVID-19. Biomed. J. 44, 235–244. doi: 10.1016/j.bj.2021.04.006. PMID: 34193390 PMC8059258

[B4] Bai C. Zhong Q. Gao G. F. (2022). Overview of SARS-CoV-2 genome-encoded proteins. Sci. China Life Sci. 65, 280–294. doi: 10.1007/s11427-021-1964-4. PMID: 34387838 PMC8362648

[B5] Bakaletz L. O. (2017). Viral-bacterial co-infections in the respiratory tract. Curr. Opin. Microbiol. 35, 30–35. doi: 10.1016/j.mib.2016.11.003. PMID: 27940028 PMC7108227

[B6] Banerjee A. K. Blanco M. R. Bruce E. A. Honson D. D. Chen L. M. Chow A. . (2020). SARS-CoV-2 disrupts splicing, translation, and protein trafficking to suppress host defenses. Cell. 183, 1325–1339.e21. doi: 10.1016/j.cell.2020.10.004. PMID: 33080218 PMC7543886

[B7] Banerjee A. Czinn S. J. Reiter R. J. Blanchard T. G . (2020). Crosstalk between endoplasmic reticulum stress and anti-viral activities: A novel therapeutic target for COVID-19. Life Sci. 255, 117842. doi: 10.1016/j.lfs.2020.117842. PMID: 32454157 PMC7245231

[B8] Belizário J. E. (2021). Immunity, virus evolution, and effectiveness of SARS-CoV-2 vaccines. Braz. J. Med. Biol. Res. 54, e10725. 33729394 10.1590/1414-431X202010725 PMC7959154

[B9] Benton D. J. Gamblin S. J. Rosenthal P. B. Skehel J. J . (2020). Structural transitions in influenza haemagglutinin at membrane fusion pH. Nature 583, 150–153. doi: 10.1038/s41586-020-2333-6. PMID: 32461688 PMC7116728

[B10] Beyerstedt S. Casaro E. B. Rangel É. B. (2021). COVID-19: angiotensin-converting enzyme 2 (ACE2) expression and tissue susceptibility to SARS-CoV-2 infection. Eur. J. Clin. Microbiol. Infect. Dis. 40, 905–919. doi: 10.1007/s10096-020-04138-6. PMID: 33389262 PMC7778857

[B11] Campbell G. R. To R. K. Hanna J. Spector S. A . (2021). SARS-CoV-2, SARS-CoV-1, and HIV-1 derived ssRNA sequences activate the NLRP3 inflammasome in human macrophages through a non-classical pathway. iScience 24, 102295. doi: 10.1016/j.isci.2021.102295. PMID: 33718825 PMC7939994

[B12] Carfì A. Bernabei R. Landi F. (2020). Persistent symptoms in patients after acute COVID-19. Jama 324, 603–605. 32644129 10.1001/jama.2020.12603 PMC7349096

[B13] Chen M. Deng H. Zhao Y. Miao X. Gu H. Bi Y. . (2022a). Toll-Like Receptor 2 modulates pulmonary inflammation and TNF-α release mediated by Mycoplasma pneumoniae. Front. Cell. Infect. Microbiol. 12, 824027. doi: 10.3389/fcimb.2022.824027. PMID: 35372108 PMC8968444

[B14] Chen X. Liu S. Goraya M. U. Maarouf M. Huang S. Chen J. L . (2018). Host immune response to influenza A virus infection. Front. Immunol. 9, 320. doi: 10.3389/fimmu.2018.00320. PMID: 29556226 PMC5845129

[B15] Chen M. Ma Y. Chang W. (2022b). SARS-coV-2 and the nucleus. Int. J. Biol. Sci. 18, 4731–4743. doi: 10.7150/ijbs.72482. PMID: 35874947 PMC9305274

[B16] Chen D. Zhao Y. G. Zhang H. (2022). Endomembrane remodeling in SARS-CoV-2 infection. Cell. Insight 1, 100031. doi: 10.1016/j.cellin.2022.100031. PMID: 37193051 PMC9112566

[B17] Chiu M. C. Li C. Liu X. Song W. Wan Z. Yu Y. . (2022). Human nasal organoids model SARS-CoV-2 upper respiratory infection and recapitulate the differential infectivity of emerging variants. mBio 13, e0194422. doi: 10.1128/mbio.01944-22. PMID: 35938726 PMC9426414

[B18] Chmura K. Bai X. Nakamura M. Kandasamy P. McGibney M. Kuronuma K. . (2008). Induction of IL-8 by Mycoplasma pneumoniae membrane in BEAS-2B cells. Am. J. Physiol. Lung Cell. Mol. Physiol. 295, L220–L230. doi: 10.1152/ajplung.90204.2008 18487355 PMC2494795

[B19] Chunlian X. Lili Z. Libin Z. Yichun X . (2025). Macrolide-resistant Mycoplasma pneumoniae is an independent risk factor for bronchial mucus plug formation in children. Eur. J. Pediatr. 184, 264. doi: 10.1007/s00431-025-06095-8. PMID: 40123017 PMC11930860

[B20] Creytens S. Pascha M. N. Ballegeer M. Saelens X. de Haan C. A. M . (2021). Influenza neuraminidase characteristics and potential as a vaccine target. Front. Immunol. 12, 786617. doi: 10.3389/fimmu.2021.786617. PMID: 34868073 PMC8635103

[B21] Cruz A. Joseph S. (2022). Interaction of the influenza A virus NS1 protein with the 5'-m7G-mRNA·eIF4E·eIF4G1 complex. Biochemistry 61, 1485–1494. doi: 10.1021/acs.biochem.2c00019. PMID: 35797022 PMC10164398

[B22] Dai J. P. Wang Q. W. Su Y. Gu L. M. Zhao Y. Chen X. X. . (2017). Emodin inhibition of influenza A virus replication and influenza viral pneumonia via the Nrf2, TLR4, p38/JNK and NF-kappaB pathways. Molecules 22. doi: 10.3390/molecules22101754. PMID: 29057806 PMC6151665

[B23] Danladi J. Sabir H. (2021). Innate immunity, inflammation activation and heat-shock protein in COVID-19 pathogenesis. J. Neuroimmunol. 358, 577632. doi: 10.1016/j.jneuroim.2021.577632. PMID: 34186336 PMC8196476

[B24] Ding X. Kambara H. Guo R. Kanneganti A. Acosta-Zaldívar M. Li J. . (2021). Inflammasome-mediated GSDMD activation facilitates escape of Candida albicans from macrophages. Nat. Commun. 12, 6699. doi: 10.1038/s41467-021-27034-9. PMID: 34795266 PMC8602704

[B25] Djomkam A. L. Z. Olwal C. O. Sala T. B. Paemka L . (2020). Commentary: SARS-CoV-2 cell entry depends on ACE2 and TMPRSS2 and is blocked by a clinically proven protease inhibitor. Front. Oncol. 10, 1448. doi: 10.3389/fonc.2020.01448. PMID: 32974166 PMC7466403

[B26] Fang Y. Liu H. Huang H. Li H. Saqi A. Qiang L. . (2020). Distinct stem/progenitor cells proliferate to regenerate the trachea, intrapulmonary airways and alveoli in COVID-19 patients. Cell Res. 30, 705–707. doi: 10.1038/s41422-020-0367-9. PMID: 32606347 PMC7325636

[B27] Flórido M. Chiu J. Hogg P. J. (2021). Influenza A virus hemagglutinin is produced in different disulfide-bonded states. Antioxid. Redox Signal. 35, 1081–1092. 33985344 10.1089/ars.2021.0033

[B28] Foo C. X. Bartlett S. Chew K. Y. Ngo M. D. Bielefeldt-Ohmann H. Arachchige B. J. . (2023). GPR183 antagonism reduces macrophage infiltration in influenza and SARS-CoV-2 infection. Eur. Respir. J. 61. doi: 10.1183/13993003.01306-2022. PMID: 36396144 PMC9686317

[B29] Frey A. Lunding L. P. Ehlers J. C. Weckmann M. Zissler U. M. Wegmann M . (2020). More than just a barrier: the immune functions of the airway epithelium in asthma pathogenesis. Front. Immunol. 11, 761. doi: 10.3389/fimmu.2020.00761. PMID: 32411147 PMC7198799

[B30] Fu Y. Z. Wang S. Y. Zheng Z. Q. Yi H. Li W. W. Xu Z. S. . (2021). SARS-CoV-2 membrane glycoprotein M antagonizes the MAVS-mediated innate antiviral response. Cell. Mol. Immunol. 18, 613–620. doi: 10.1038/s41423-020-00571-x. PMID: 33110251 PMC7588591

[B31] Ganjian H. Rajput C. Elzoheiry M. Sajjan U . (2020). Rhinovirus and innate immune function of airway epithelium. Front. Cell. Infect. Microbiol. 10, 277. doi: 10.3389/fcimb.2020.00277. PMID: 32637363 PMC7316886

[B32] Gao L. Sun Y. (2024). Laboratory diagnosis and treatment of Mycoplasma pneumoniae infection in children: a review. Ann. Med. 56, 2386636. doi: 10.1080/07853890.2024.2386636. PMID: 39097794 PMC11299444

[B33] Gavaud A. Holub M. Asquier-Khati A. Faure K. Leautez-Nainville S. Le Moal G. . (2025). Mycoplasma pneumoniae infection in adult inpatients during the 2023–24 outbreak in France (MYCADO): a national, retrospective, observational study. Lancet Infect. Dis. 25, 801–812. doi: 10.1016/s1473-3099(24)00805-3. PMID: 39986287

[B34] Georgakopoulou V. E. Lempesis I. G. Sklapani P. Trakas N. Spandidos D. A . (2024). Exploring the pathogenetic mechanisms of Mycoplasmapneumoniae (review). Exp. Ther. Med. 28, 271. doi: 10.3892/etm.2024.12559. PMID: 38765654 PMC11097136

[B35] Goncheva M. I. Conceicao C. Tuffs S. W. Lee H. M. Quigg-Nicol M. Bennet I. . (2020). Staphylococcus aureus Lipase 1 enhances influenza A virus replication. mBio 11. doi: 10.1128/mbio.00975-20. PMID: 32636247 PMC7343990

[B36] Gonzalez-Orozco M. Tseng H. C. Hage A. Xia H. Behera P. Afreen K. . (2024). TRIM7 ubiquitinates SARS-CoV-2 membrane protein to limit apoptosis and viral replication. Nat. Commun. 15, 10438. doi: 10.1038/s41467-024-54762-5. PMID: 39616206 PMC11608229

[B37] Gordon D. E. Jang G. M. Bouhaddou M. Xu J. Obernier K. White K. M. . (2020). A SARS-CoV-2 protein interaction map reveals targets for drug repurposing. Nature 583, 459–468. doi: 10.1038/s41586-020-2286-9. PMID: 32353859 PMC7431030

[B38] Grigoryan L. Pulendran B. (2020). The immunology of SARS-CoV-2 infections and vaccines. Semin. Immunol. 50, 101422. doi: 10.1016/j.smim.2020.101422. PMID: 33262067 PMC7670910

[B39] Gu Y. Zuo X. Zhang S. Ouyang Z. Jiang S. Wang F. . (2021). The mechanism behind influenza virus cytokine storm. Viruses 13. doi: 10.3390/v13071362. PMID: 34372568 PMC8310017

[B40] Guney C. Akar F. (2021). Epithelial and endothelial expressions of ACE2: SARS-CoV-2 entry routes. J. Pharm. Pharm. Sci. 24, 84–93. doi: 10.18433/jpps31455. PMID: 33626315

[B41] Guo Z. Banas V. S. He Y. Weiland E. Xu J. Tan Y. . (2025). Ciliated cells promote high infectious potential of influenza A virus through the efficient intracellular activation of hemagglutinin. J. Virol. 99, e0068525. doi: 10.1101/2025.04.30.651529. PMID: 40882005 PMC12456007

[B42] Ha D. P. Van Krieken R. Carlos A. J Lee A. S. (2020). The stress-inducible molecular chaperone GRP78 as potential therapeutic target for coronavirus infection. J. Infect. 81, 452–482. doi: 10.1016/j.jinf.2020.06.017. PMID: 32535155 PMC7289740

[B43] Hartshorn K. L. (2020). Innate immunity and influenza A virus pathogenesis: lessons for COVID-19. Front. Cell. Infect. Microbiol. 10, 563850. doi: 10.3389/fcimb.2020.563850. PMID: 33194802 PMC7642997

[B44] Hatabu H. Kaye K. M. Christiani D. C. (2023). Viral infection, pulmonary fibrosis, and long COVID. Am. J. Respir. Crit. Care Med. 207, 647–649. doi: 10.1164/rccm.202211-2121ed. PMID: 36470237 PMC10037483

[B45] Hewitt R. J. Lloyd C. M. (2021). Regulation of immune responses by the airway epithelial cell landscape. Nat. Rev. Immunol. 21, 347–362. doi: 10.1038/s41577-020-00477-9. PMID: 33442032 PMC7804588

[B46] Hoffmann M. Kleine-Weber H. Schroeder S. Krüger N. Herrler T. Erichsen S. . (2020). SARS-CoV-2 cell entry depends on ACE2 and TMPRSS2 and is blocked by a clinically proven protease inhibitor. Cell. 181, 271–280.e8. doi: 10.1016/j.cell.2020.02.052. PMID: 32142651 PMC7102627

[B47] Hu J. Ye Y. Chen X. Xiong L. Xie W. Liu P . (2022). Insight into the pathogenic mechanism of Mycoplasma pneumoniae. Curr. Microbiol. 80, 14. doi: 10.1007/s00284-022-03103-0. PMID: 36459213 PMC9716528

[B48] Hui X. Cao L. Xu T. Zhao L. Huang K. Zou Z. . (2022). PSMD12-mediated M1 ubiquitination of influenza A virus at K102 regulates viral replication. J. Virol. 96, e0078622. doi: 10.1128/jvi.00786-22. PMID: 35861516 PMC9364790

[B49] Hui K. P. Y. Ho J. C. W. Cheung M. C. Ng K. C. Ching R. H. H. Lai K. L. . (2022). SARS-CoV-2 Omicron variant replication in human bronchus and lung ex vivo. Nature 603, 715–720. doi: 10.1038/s41586-022-04479-6. PMID: 35104836

[B50] Hunter C. A. Jones S. A. (2015). IL-6 as a keystone cytokine in health and disease. Nat. Immunol. 16, 448–457. doi: 10.1038/ni.3153. PMID: 25898198

[B51] Iannuzo N. Dy A. B. C. Guerra S. Langlais P. R. Ledford J. G . (2023). The impact of CC16 on pulmonary epithelial-driven host responses during Mycoplasma pneumoniae infection in mouse tracheal epithelial cells. Cells 12. doi: 10.3390/cells12151984. PMID: 37566063 PMC10416898

[B52] Invernizzi R. Lloyd C. M. Molyneaux P. L. (2020). Respiratory microbiome and epithelial interactions shape immunity in the lungs. Immunology 160, 171–182. doi: 10.1111/imm.13195. PMID: 32196653 PMC7218407

[B53] Islamuddin M. Mustfa S. A. Ullah S. Omer U. Kato K. Parveen S. (2022). Innate immune response and inflammasome activation during SARS-CoV-2 infection. Inflammation 45, 1849–1863. doi: 10.1007/s10753-022-01651-y. PMID: 35953688 PMC9371632

[B54] Ivanova T. Mariienko Y. Mehterov N. Kazakova M. Sbirkov Y. Todorova K. . (2023). Autophagy and SARS-CoV-2-old players in new games. Int. J. Mol. Sci. 24. doi: 10.3390/ijms24097734. PMID: 37175443 PMC10178552

[B55] Iverson A. R. Boyd K. L. McAuley J. L. Plano L. R. Hart M. E. McCullers J. A. (2011). Influenza virus primes mice for pneumonia from Staphylococcus aureus. J. Infect. Dis. 203, 880–888. doi: 10.1093/infdis/jiq113. PMID: 21278211 PMC3071123

[B56] Iwasaki A. Pillai P. S. (2014). Innate immunity to influenza virus infection. Nat. Rev. Immunol. 14, 315–328. doi: 10.1038/nri3665. PMID: 24762827 PMC4104278

[B57] Jiang Z. Li S. Zhu C. Zhou R. Leung P. H. M . (2021). Mycoplasma pneumoniae infections: pathogenesis and vaccine development. Pathogens 10. doi: 10.3390/pathogens10020119. PMID: 33503845 PMC7911756

[B58] Jiao P. Fan W. Ma X. Lin R. Zhao Y. Li Y. . (2023). SARS-CoV-2 nonstructural protein 6 triggers endoplasmic reticulum stress-induced autophagy to degrade STING1. Autophagy 19, 3113–3131. doi: 10.1080/15548627.2023.2238579. PMID: 37482689 PMC10621274

[B59] Kaivola J. Nyman T. A. Matikainen S. (2021). Inflammasomes and SARS-coV-2 infection. Viruses 13. doi: 10.3390/v13122513. PMID: 34960782 PMC8706865

[B60] Kalil A. C. Thomas P. G. (2019). Influenza virus-related critical illness: pathophysiology and epidemiology. Crit. Care 23, 258. doi: 10.1186/s13054-019-2539-x. PMID: 31324202 PMC6642581

[B61] Karaaslan A. Çetin C. Akın Y. Demir Tekol S. Söbü E. Demirhan R. (2021). Coinfection in SARS-CoV-2 infected children patients. J. Infect. Dev. Ctries 15, 761–765. doi: 10.3855/jidc.14314. PMID: 34242183

[B62] Kuriakose T. Kanneganti T. D. (2017). Regulation and functions of NLRP3 inflammasome during influenza virus infection. Mol. Immunol. 86, 56–64. doi: 10.1016/j.molimm.2017.01.023. PMID: 28169000 PMC5453821

[B63] Kuriakose T. Man S. M. Malireddi R. K. Karki R. Kesavardhana S. Place D. E. . (2016). ZBP1/DAI is an innate sensor of influenza virus triggering the NLRP3 inflammasome and programmed cell death pathways. Sci. Immunol. 1. doi: 10.1126/sciimmunol.aag2045. PMID: 27917412 PMC5131924

[B64] Lee J. H. Choi M. Jung Y. Lee S. K. Lee C. S. Kim J. . (2021). A novel rapid detection for SARS-CoV-2 spike 1 antigens using human angiotensin converting enzyme 2 (ACE2). Biosens. Bioelectron. 171, 112715. doi: 10.1016/j.bios.2020.112715. PMID: 33099241 PMC7560266

[B65] Lei X. Dong X. Ma R. Wang W. Xiao X. Tian Z. . (2020). Activation and evasion of type I interferon responses by SARS-CoV-2. Nat. Commun. 11, 3810. doi: 10.1038/s41467-020-17665-9. PMID: 32733001 PMC7392898

[B66] LeMessurier K. S. Tiwary M. Morin N. P. Samarasinghe A. E . (2020). Respiratory barrier as a safeguard and regulator of defense against influenza A virus and Streptococcus pneumoniae. Front. Immunol. 11, 3. doi: 10.3389/fimmu.2020.00003. PMID: 32117216 PMC7011736

[B67] Li X. Han B. Chen Y. Lu H . (2024). Strengthening medical facility responses to respiratory infectious diseases: global trends, challenges, and innovations post-COVID-19. Biosci. Trends 18, 404–408. doi: 10.5582/bst.2024.01197. PMID: 39414464

[B68] Li X. Huang C. Rai K. R. Xu Q . (2025a). Innate immune role of IL-6 in influenza a virus pathogenesis. Front. Cell. Infect. Microbiol. 15, 1605446. doi: 10.3389/fcimb.2025.1605446. PMID: 40692679 PMC12277322

[B69] Li W. Li M. Ou G. (2020). COVID-19, cilia, and smell. FEBS J. 287, 3672–3676. doi: 10.1111/febs.15491. PMID: 32692465 PMC7426555

[B70] Li F. Li J. Wang P. H. Yang N. Huang J. Ou J. . (2021). SARS-CoV-2 spike promotes inflammation and apoptosis through autophagy by ROS-suppressed PI3K/AKT/mTOR signaling. Biochim. Biophys. Acta Mol. Basis Dis. 1867, 166260. doi: 10.1016/j.bbadis.2021.166260. PMID: 34461258 PMC8390448

[B71] Li N. Ren A. Wang X. Fan X. Zhao Y. Gao G. F. . (2015). Influenza viral neuraminidase primes bacterial coinfection through TGF-β-mediated expression of host cell receptors. Proc. Natl. Acad. Sci. U.S.A. 112, 238–243. doi: 10.1073/pnas.1414422112. PMID: 25535343 PMC4291618

[B72] Li B. Wang D. Zhang C. Wang Y. Huang Z. Yang L. . (2024). Role of respiratory system microbiota in development of lung cancer and clinical application. Imeta 3, e232. doi: 10.1002/imt2.232. PMID: 39429871 PMC11488069

[B73] Li S. Xue G. Zhao H. Feng Y. Yan C. Cui J. . (2019). The Mycoplasma pneumoniae HapE alters the cytokine profile and growth of human bronchial epithelial cells. Biosci. Rep. 39. doi: 10.1042/bsr20182201. PMID: 30573530 PMC6340952

[B74] Li X. Zeng Q. Liu C. Yi X. Luo H. Tong Q. . (2025b). The immune modulatory role of surfactants in Mycoplasma pneumoniae infection. J. Inflamm. Res. 18, 2909–2922. doi: 10.2147/jir.s507526. PMID: 40034686 PMC11873027

[B75] Li L. Zhang Y. Zhao L. Shi Y . (2025). C-reactive protein-induced injury in Mycoplasma pneumoniae-infected lung epithelial cells is mediated by the P38 MAPK/mitochondrial apoptosis pathway. Microbiol. Spectr. 13, e0162624. doi: 10.1128/spectrum.01626-24. PMID: 39932324 PMC11878036

[B76] Linfield D. T. Raduka A. Aghapour M. Rezaee F . (2021). Airway tight junctions as targets of viral infections. Tissue Barriers 9, 1883965. doi: 10.1080/21688370.2021.1883965. PMID: 33632074 PMC8078511

[B77] Liu X. Kimmey J. M. Matarazzo L. de Bakker V. Van Maele L. Sirard J. C. . (2021). Exploration of bacterial bottlenecks and Streptococcus pneumoniae pathogenesis by CRISPRi-Seq. Cell Host Microbe 29, 107–120.e6. doi: 10.1016/j.chom.2020.10.001. PMID: 33120116 PMC7855995

[B78] Liu Y. Li J. Lu X. Zhen S. Huo J . (2022). Toll-Like Receptor 4 exacerbates Mycoplasma pneumoniaevia promoting transcription factor EB-mediated autophagy. Contrast Media Mol. Imaging 2022, 3357694. doi: 10.1155/2022/3357694. PMID: 35965629 PMC9357725

[B79] Low Z. Y. Zabidi N. Z. Yip A. J. W. Puniyamurti A. Chow V. T. K. Lal S. K. . (2022). SARS-CoV-2 non-structural proteins and their roles in host immune evasion. Viruses 14. doi: 10.3390/v14091991. PMID: 36146796 PMC9506350

[B80] Lubkin A. Bernard-Raichon L. DuMont A. L. Valero Jimenez A. M. Putzel G. G. Gago J. . (2024). SARS-CoV-2 infection predisposes patients to coinfection with Staphylococcus aureus. mBio 15, e0166724. doi: 10.1128/mbio.01667-24. PMID: 39037272 PMC11323729

[B81] Luo H. He J. Qin L. Chen Y. Chen L. Li R. . (2021). Mycoplasma pneumoniae lipids license TLR-4 for activation of NLRP3 inflammasome and autophagy to evoke a proinflammatory response. Clin. Exp. Immunol. 203, 66–79. doi: 10.1111/cei.13510. PMID: 32894580 PMC7744503

[B82] Ma C. Sacco M. D. Hurst B. Townsend J. A. Hu Y. Szeto T. . (2020). Boceprevir, GC-376, and calpain inhibitors II, XII inhibit SARS-CoV-2 viral replication by targeting the viral main protease. Cell Res. 30, 678–692. doi: 10.1038/s41422-020-0356-z. PMID: 32541865 PMC7294525

[B83] Ma H. Zhu Z. Lin H. Wang S. Zhang P. Li Y. . (2021). Pyroptosis of syncytia formed by fusion of SARS-CoV-2 spike and ACE2-expressing cells. Cell. Discov. 7, 73. doi: 10.1038/s41421-021-00310-0. PMID: 34429403 PMC8384103

[B84] Ma X. Zou F. Yu F. Li R. Yuan Y. Zhang Y. . (2020). Nanoparticle vaccines based on the receptor binding domain (RBD) and heptad repeat (HR) of SARS-CoV-2 elicit robust protective immune responses. Immunity 53, 1315–1330.e9. doi: 10.1016/j.immuni.2020.11.015. PMID: 33275896 PMC7687490

[B85] Marchi E. Hinks T. S. C. Richardson M. Khalfaoui L. Symon F. A. Rajasekar P. . (2024). The effects of inhaled corticosteroids on healthy airways. Allergy 79, 1831–1843. doi: 10.1183/13993003.congress-2024.oa2777. PMID: 38686450 PMC7616167

[B86] Mehta P. McAuley D. F. Brown M. Sanchez E. Tattersall R. S. Manson J. J. (2020). COVID-19: consider cytokine storm syndromes and immunosuppression. Lancet 395, 1033–1034. doi: 10.1016/s0140-6736(20)30628-0. PMID: 32192578 PMC7270045

[B87] Meng B. Datir R. Choi J. Bradley J. R. Smith K. G. C. Lee J. H. . (2022). SARS-CoV-2 spike N-terminal domain modulates TMPRSS2-dependent viral entry and fusogenicity. Cell Rep. 40, 111220. doi: 10.1016/j.celrep.2022.111220. PMID: 35963244 PMC9346021

[B88] Metzger D. W. Sun K. (2013). Immune dysfunction and bacterial coinfections following influenza. J. Immunol. 191, 2047–2052. doi: 10.4049/jimmunol.1301152. PMID: 23964104 PMC3760235

[B89] Mifsud E. J. Kuba M. Barr I. G. (2021). Innate immune responses to influenza virus infections in the upper respiratory tract. Viruses 13. doi: 10.3390/v13102090. PMID: 34696520 PMC8541359

[B90] Miorin L. Kehrer T. Sanchez-Aparicio M. T. Zhang K. Cohen P. Patel R. S. . (2020). SARS-CoV-2 Orf6 hijacks Nup98 to block STAT nuclear import and antagonize interferon signaling. Proc. Natl. Acad. Sci. U.S.A. 117, 28344–28354. doi: 10.1073/pnas.2016650117. PMID: 33097660 PMC7668094

[B91] Mirzaei R. Goodarzi P. Asadi M. Soltani A. Aljanabi H. A. A. Jeda A. S. . (2020). Bacterial co-infections with SARS-cov-2. IUBMB Life 72, 2097–2111. doi: 10.1002/iub.2356 32770825 PMC7436231

[B92] Muus C. Luecken M. D. Eraslan G. Sikkema L. Waghray A. Heimberg G. . (2021). Single-cell meta-analysis of SARS-CoV-2 entry genes across tissues and demographics. Nat. Med. 27, 546–559. doi: 10.1038/s41591-020-01227-z. PMID: 33654293 PMC9469728

[B93] Nakamura S. Davis K. M. Weiser J. N. (2011). Synergistic stimulation of type I interferons during influenza virus coinfection promotes Streptococcus pneumoniae colonization in mice. J. Clin. Invest. 121, 3657–3665. doi: 10.1172/jci57762. PMID: 21841308 PMC3163966

[B94] Naqvi K. F. Mazzone S. B. Shiloh M. U. (2023). Infectious and inflammatory pathways to cough. Annu. Rev. Physiol. 85, 71–91. doi: 10.1146/annurev-physiol-031422-092315. PMID: 36170660 PMC9918720

[B95] Nelli R. K. Harm T. A. Siepker C. Groeltz-Thrush J. M. Jones B. Twu N. C. . (2024). Sialic acid receptor specificity in mammary gland of dairy cattle infected with highly pathogenic avian influenza A(H5N1) virus. Emerg. Infect. Dis. 30, 1361–1373. doi: 10.3201/eid3007.240689. PMID: 38861554 PMC11210646

[B96] Nguyen N. N. Nguyen Y. N. Hoang V. T. Million M. Gautret P . (2023). SARS-CoV-2 reinfection and severity of the disease: a systematic review and meta-analysis. Viruses 15. doi: 10.3390/v15040967. PMID: 37112949 PMC10145185

[B97] Nypaver C. Dehlinger C. Carter C. (2021). Influenza and influenza vaccine: a review. J. Midwifery Womens Health 66, 45–53. doi: 10.1111/jmwh.13203. PMID: 33522695 PMC8014756

[B98] Onufer A. P. Mell J. C. Cort L. Rao A. Mdluli N. V. Carey A. J. (2025). Influenza virus-induced type I interferons disrupt alveolar epithelial repair and tight junction integrity in the developing lung. Mucosal Immunol. 18, 607–619. doi: 10.1016/j.mucimm.2025.02.002. PMID: 39984053 PMC12710878

[B99] Ou X. Liu Y. Lei X. Li P. Mi D. Ren L. . (2020). Characterization of spike glycoprotein of SARS-CoV-2 on virus entry and its immune cross-reactivity with SARS-CoV. Nat. Commun. 11, 1620. doi: 10.1038/s41467-020-15562-9. PMID: 32221306 PMC7100515

[B100] Pan P. Shen M. Yu Z. Ge W. Chen K. Tian M. . (2021). SARS-CoV-2 N protein promotes NLRP3 inflammasome activation to induce hyperinflammation. Nat. Commun. 12, 4664. doi: 10.1038/s41467-021-25015-6. PMID: 34341353 PMC8329225

[B101] Pandey K. P. Zhou Y. (2022). Influenza A virus infection activates NLRP3 inflammasome through trans-Golgi network dispersion. Viruses 14. doi: 10.3390/v14010088. PMID: 35062292 PMC8778788

[B102] Parasher A. (2021). COVID-19: Current understanding of its pathophysiology, clinical presentation and treatment. Postgrad. Med. J. 97, 312–320. doi: 10.1136/postgradmedj-2020-138577. PMID: 32978337 PMC10017004

[B103] Park M. D. (2020). Macrophages: a trojan horse in COVID-19? Nat. Rev. Immunol. 20, 351. doi: 10.1038/s41577-020-0317-2. PMID: 32303696 PMC7186930

[B104] Peluso M. J. Deeks S. G. (2024). Mechanisms of long COVID and the path toward therapeutics. Cell. 187, 5500–5529. doi: 10.1016/j.cell.2024.07.054. PMID: 39326415 PMC11455603

[B105] Pitré E. Bisht K. Remick K. A. Ghorbani A. Yewdell J. W. Elshina E. . (2025). A two-step mechanism for RIG-I activation by influenza virus mvRNAs. Sci. Adv. 11, eadw8034. doi: 10.1126/sciadv.adw8034 40779639 PMC12333679

[B106] Qin L. Liu L. Wu Y. Chen Y. Wu Y. Luo H. . (2022). Mycoplasma pneumoniae downregulates RECK to promote matrix metalloproteinase-9 secretion by bronchial epithelial cells. Virulence 13, 1270–1284. doi: 10.1080/21505594.2022.2101746. PMID: 35892136 PMC9336473

[B107] Rajah M. M. Bernier A. Buchrieser J. Schwartz O . (2022). The mechanism and consequences of SARS-CoV-2 spike-mediated fusion and syncytia formation. J. Mol. Biol. 434, 167280. doi: 10.1016/j.jmb.2021.167280. PMID: 34606831 PMC8485708

[B108] Ramasamy K. Balasubramanian S. Kirkpatrick A. Szabo D. Pandranki L. Baseman J. B. . (2021). Mycoplasma pneumoniae CARDS toxin exploits host cell endosomal acidic pH and vacuolar ATPase proton pump to execute its biological activities. Sci. Rep. 11, 11571. doi: 10.1038/s41598-021-90948-3. PMID: 34078958 PMC8172646

[B109] Rashid F. Xie Z. Li M. Xie Z. Luo S. Xie L. (2023). Roles and functions of IAV proteins in host immune evasion. Front. Immunol. 14, 1323560. doi: 10.3389/fimmu.2023.1323560. PMID: 38152399 PMC10751371

[B110] Rashid F. Xie Z. Suleman M. Shah A. Khan S. Luo S. (2022). Roles and functions of SARS-CoV-2 proteins in host immune evasion. Front. Immunol. 13, 940756. doi: 10.3389/fimmu.2022.940756. PMID: 36003396 PMC9394213

[B111] Rasmussen S. A. Smulian J. C. Lednicky J. A. Wen T. S. Jamieson D. J . (2020). Coronavirus disease 2019 (COVID-19) and pregnancy: What obstetricians need to know. Am. J. Obstet. Gynecol 222, 415–426. doi: 10.1097/01.aoa.0000719440.84472.52. PMID: 32105680 PMC7093856

[B112] Ren Y. Wang A. Fang Y. Shu T. Wu D. Wang C. . (2021). SARS-CoV-2 membrane glycoprotein M triggers apoptosis with the assistance of nucleocapsid protein N in cells. Front. Cell. Infect. Microbiol. 11, 706252. doi: 10.3389/fcimb.2021.706252. PMID: 34513728 PMC8425412

[B113] Robinot R. Hubert M. de Melo G. D. Lazarini F. Bruel T. Smith N. . (2021). SARS-CoV-2 infection induces the dedifferentiation of multiciliated cells and impairs mucociliary clearance. Nat. Commun. 12, 4354. doi: 10.1038/s41467-021-24521-x. PMID: 34272374 PMC8285531

[B114] Rossman J. S. Lamb R. A. (2011). Influenza virus assembly and budding. Virology 411, 229–236. doi: 10.1016/j.virol.2010.12.003. PMID: 21237476 PMC3086653

[B115] Salvi V. Nguyen H. O. Sozio F. Schioppa T. Gaudenzi C. Laffranchi M. . (2021). SARS-CoV-2-associated ssRNAs activate inflammation and immunity via TLR7/8. JCI Insight 6. doi: 10.1101/2021.04.15.439839. PMID: 34375313 PMC8492321

[B116] Savan R. Gale M. (2023). Innate immunity and interferon in SARS-CoV-2 infection outcome. Immunity 56, 1443–1450. doi: 10.1016/j.immuni.2023.06.018. PMID: 37437537 PMC10361255

[B117] Schumacher M. Nicholson P. Stoffel M. H. Chandran S. D'Mello A. Ma L. . (2019). Evidence for the cytoplasmic localization of the L-α-glycerophosphate oxidase in members of the "Mycoplasma mycoides cluster. Front. Microbiol. 10, 1344. doi: 10.3389/fmicb.2019.01344. PMID: 31275271 PMC6593217

[B118] Sheng Y. H. Hasnain S. Z. (2022). Mucus and mucins: the underappreciated host defence system. Front. Cell. Infect. Microbiol. 12, 856962. doi: 10.3389/fcimb.2022.856962. PMID: 35774401 PMC9238349

[B119] Short K. R. Kasper J. van der Aa S. Andeweg A. C. Zaaraoui-Boutahar F. Goeijenbier M. . (2016). Influenza virus damages the alveolar barrier by disrupting epithelial cell tight junctions. Eur. Respir. J. 47, 954–966. doi: 10.1183/13993003.01282-2015. PMID: 26743480

[B120] Shuai H. Chan J. F. Hu B. Chai Y. Yuen T. T. Yin F. . (2022). Attenuated replication and pathogenicity of SARS-CoV-2 B.1.1.529 Omicron. Nature 603, 693–699. doi: 10.1038/s41586-022-04442-5. PMID: 35062016

[B121] Sicari D. Chatziioannou A. Koutsandreas T. Sitia R. Chevet E . (2020). Role of the early secretory pathway in SARS-CoV-2 infection. J. Cell Biol. 219. doi: 10.1083/jcb.202006005. PMID: 32725137 PMC7480111

[B122] Siu K. L. Yuen K. S. Castaño-Rodriguez C. Ye Z. W. Yeung M. L. Fung S. Y. . (2019). Severe acute respiratory syndrome coronavirus ORF3a protein activates the NLRP3 inflammasome by promoting TRAF3-dependent ubiquitination of ASC. FASEB J. 33, 8865–8877. doi: 10.1096/fj.201802418r. PMID: 31034780 PMC6662968

[B123] Smith A. M. Adler F. R. Ribeiro R. M. Gutenkunst R. N. McAuley J. L. McCullers J. A. . (2013). Kinetics of coinfection with influenza A virus and Streptococcus pneumoniae. PLoS Pathog. 9, e1003238. doi: 10.1371/journal.ppat.1003238. PMID: 23555251 PMC3605146

[B124] Song Z. Han C. Luo G. Jia G. Wang X. Zhang B. (2024). Yinqin Qingfei granules alleviate Mycoplasma pneumoniae pneumonia via inhibiting NLRP3 inflammasome-mediated macrophage pyroptosis. Front. Pharmacol. 15, 1437475. doi: 10.3389/fphar.2024.1437475. PMID: 39257401 PMC11383775

[B125] Speaks S. McFadden M. I. Zani A. Solstad A. Leumi S. Roettger J. E. . (2024). Gasdermin D promotes influenza virus-induced mortality through neutrophil amplification of inflammation. Nat. Commun. 15, 2751. doi: 10.1038/s41467-024-47067-0. PMID: 38553499 PMC10980740

[B126] Strobelt R. Broennimann K. Adler J. Shaul Y . (2023). SARS-CoV-2 Omicron specific mutations affecting infectivity, fusogenicity, and partial TMPRSS2-independency. Viruses 15. doi: 10.3390/v15051129. PMID: 37243215 PMC10223509

[B127] Strohmeier S. Amanat F. Carreño J. M. Krammer F . (2022). Monoclonal antibodies targeting the influenza virus N6 neuraminidase. Front. Immunol. 13, 944907. doi: 10.3389/fimmu.2022.944907. PMID: 35967389 PMC9363587

[B128] Su X. You X. Luo H. Liang K. Chen L. Tian W. . (2021). Community-acquired respiratory distress syndrome toxin: Unique exotoxin for M. pneumoniae. Front. Microbiol. 12, 766591. doi: 10.3389/fmicb.2021.766591. PMID: 34867898 PMC8640204

[B129] Sun B. Ling Y. Li J. Ma L. Jie Z. Luo H. . (2025). Advances in adhesion-related pathogenesis in Mycoplasma pneumoniae infection. Front. Microbiol. 16, 1613760. doi: 10.3389/fmicb.2025.1613760. PMID: 40771685 PMC12325244

[B130] Sun X. Liu Y. Huang Z. Xu W. Hu W. Yi L. . (2022). SARS-CoV-2 non-structural protein 6 triggers NLRP3-dependent pyroptosis by targeting ATP6AP1. Cell Death Differ. 29, 1240–1254. doi: 10.1038/s41418-021-00916-7. PMID: 34997207 PMC9177730

[B131] Sureda A. Alizadeh J. Nabavi S. F. Berindan-Neagoe I. Cismaru C. A. Jeandet P. . (2020). Endoplasmic reticulum as a potential therapeutic target for covid-19 infection management? Eur. J. Pharmacol. 882, 173288. doi: 10.1016/j.ejphar.2020.173288. PMID: 32561291 PMC7297682

[B132] Suzuki R. Yamasoba D. Kimura I. Wang L. Kishimoto M. Ito J. . (2022). Attenuated fusogenicity and pathogenicity of SARS-CoV-2 Omicron variant. Nature 603, 700–705. doi: 10.1038/s41586-022-04462-1. PMID: 35104835 PMC8942852

[B133] Takeda M. (2022). Proteolytic activation of SARS-CoV-2 spike protein. Microbiol. Immunol. 66, 15–23. doi: 10.1111/1348-0421.12945. PMID: 34561887 PMC8652499

[B134] Tan X. Cai K. Li J. Yuan Z. Chen R. Xiao H. . (2023). Coronavirus subverts ER-phagy by hijacking FAM134B and ATL3 into p62 condensates to facilitate viral replication. Cell Rep. 42, 112286. doi: 10.1016/j.celrep.2023.112286. PMID: 36952345 PMC9998290

[B135] Tan K. S. Liu J. Andiappan A. K. Lew Z. Z. R. He T. T. Ong H. H. . (2025). Unique immune and other responses of human nasal epithelial cells infected with H5N1 avian influenza virus compared to seasonal human influenza A and B viruses. Emerg. Microbes Infect. 14, 2484330. doi: 10.1080/22221751.2025.2484330. PMID: 40126073 PMC11980200

[B136] Upadhyay M. Gupta S. (2022). Endoplasmic reticulum secretory pathway: Potential target against SARS-CoV-2. Virus Res. 320, 198897. doi: 10.1016/j.virusres.2022.198897. PMID: 35988898 PMC9387115

[B137] Vaswani C. M. Varkouhi A. K. Gupta S. Ektesabi A. M. Tsoporis J. N. Yousef S. . (2023). Preventing occludin tight-junction disruption via inhibition of microRNA-193b-5p attenuates viral load and influenza-induced lung injury. Mol. Ther. 31, 2681–2701. doi: 10.1016/j.ymthe.2023.06.011. PMID: 37340634 PMC10491994

[B138] Vaz de Paula C. B. Nagashima S. Liberalesso V. Collete M. da Silva F. P. G. Oricil A. G. G. . (2021). COVID-19: Immunohistochemical analysis of TGF-β signaling pathways in pulmonary fibrosis. Int. J. Mol. Sci. 23. doi: 10.3390/ijms23010168. PMID: 35008594 PMC8745764

[B139] Vizarraga D. Kawamoto A. Matsumoto U. Illanes R. Pérez-Luque R. Martín J. . (2020). Immunodominant proteins P1 and P40/P90 from human pathogen Mycoplasma pneumoniae. Nat. Commun. 11, 5188. doi: 10.1038/s41467-020-18777-y. PMID: 33057023 PMC7560827

[B140] Waites K. B. Talkington D. F. (2004). Mycoplasma pneumoniae and its role as a human pathogen. Clin. Microbiol. Rev. 17, 697–728. doi: 10.1017/cbo9781139855952.188. PMID: 15489344 PMC523564

[B141] Waites K. B. Xiao L. Liu Y. Balish M. F. Atkinson T. P . (2017). Mycoplasma pneumoniae from the respiratory tract and beyond. Clin. Microbiol. Rev. 30, 747–809. doi: 10.1128/cmr.00114-16. PMID: 28539503 PMC5475226

[B142] Wang C. Wen J. Yan Z. Zhou Y. Gong Z. Luo Y. . (2024). Suppressing neutrophil itaconate production attenuates Mycoplasma pneumoniae pneumonia. PLoS Pathog. 20, e1012614. doi: 10.1371/journal.ppat.1012614. PMID: 39499730 PMC11567624

[B143] Wang R. Zhu Y. Ren C. Yang S. Tian S. Chen H. . (2021). Influenza A virus protein PB1-F2 impairs innate immunity by inducing mitophagy. Autophagy 17, 496–511. doi: 10.1080/15548627.2020.1725375. PMID: 32013669 PMC8007153

[B144] Wang Chau C. Sugimura R. (2022). Locked in a pro-inflammatory state. Elife 11. doi: 10.7554/elife.80699. PMID: 35796529 PMC9262384

[B145] Wei F. Gao C. Wang Y. (2022). The role of influenza A virus-induced hypercytokinemia. Crit. Rev. Microbiol. 48, 240–256. doi: 10.1080/1040841x.2021.1960482. PMID: 34353210

[B146] Wendisch D. Dietrich O. Mari T. von Stillfried S. Ibarra I. L. Mittermaier M. . (2021). SARS-CoV-2 infection triggers profibrotic macrophage responses and lung fibrosis. Cell. 184, 6243–6261.e27. doi: 10.1016/j.cell.2021.11.033. PMID: 34914922 PMC8626230

[B147] Wu N. C. Ellebedy A. H. (2024). Targeting neuraminidase: the next frontier for broadly protective influenza vaccines. Trends Immunol. 45, 11–19. doi: 10.1016/j.it.2023.11.001. PMID: 38103991 PMC10841738

[B148] Xia H. Cao Z. Xie X. Zhang X. Chen J. Y. Wang H. . (2020). Evasion of type I interferon by SARS-coV-2. Cell Rep. 33, 108234. doi: 10.1016/j.celrep.2020.108234. PMID: 32979938 PMC7501843

[B149] Xu H. Akinyemi I. A. Chitre S. A. Loeb J. C. Lednicky J. A. McIntosh M. T. . (2022). SARS-CoV-2 viroporin encoded by ORF3a triggers the NLRP3 inflammatory pathway. Virology 568, 13–22. doi: 10.1016/j.virol.2022.01.003. PMID: 35066302 PMC8762580

[B150] Xu N. Fan L. Li L. Guo Y . (2024). Exploring the pathogenicity of Mycoplasma pneumoniae: Focus on community-acquired respiratory distress syndrome toxins. Microb. Pathog. 195, 106865. doi: 10.1016/j.micpath.2024.106865. PMID: 39153578

[B151] Xue Y. Wang M. Han H. (2023). Interaction between alveolar macrophages and epithelial cells during Mycoplasma pneumoniae infection. Front. Cell. Infect. Microbiol. 13, 1052020. doi: 10.3389/fcimb.2023.1052020. PMID: 37113130 PMC10126420

[B152] Yamamoto T. Kida Y. Kuwano K. (2019). Mycoplasma pneumoniae protects infected epithelial cells from hydrogen peroxide-induced cell detachment. Cell. Microbiol. 21, e13015. doi: 10.1111/cmi.13015. PMID: 30702185

[B153] Yamamoto T. Kida Y. Sakamoto Y. Kuwano K . (2017). Mpn491, a secreted nuclease of Mycoplasma pneumoniae, plays a critical role in evading killing by neutrophil extracellular traps. Cell. Microbiol. 19. doi: 10.1111/cmi.12666. PMID: 27603754

[B154] Yang X. Liao D. Huang Y. Li C. Li Y. Deng Z. . (2025). CCL20 expression via AKT-ERK1/2-AP1 pathway in Mycoplasma pneumoniae infection: Implications for EMT and cell migration. J. Inflamm. Res. 18, 5727–5739. doi: 10.2147/jir.s512408. PMID: 40322529 PMC12047387

[B155] Yang Y. Wu Y. Meng X. Wang Z. Younis M. Liu Y. . (2022). SARS-CoV-2 membrane protein causes the mitochondrial apoptosis and pulmonary edema via targeting BOK. Cell Death Differ. 29, 1395–1408. doi: 10.1038/s41418-022-00928-x. PMID: 35022571 PMC8752586

[B156] Yao X. H. Li T. Y. He Z. C. Ping Y. F. Liu H. W. Yu S. C. . (2020). A pathological report of three COVID-19 cases by minimal invasive autopsies. Zhonghua Bing Li Xue Za Zhi 49, 411–417. doi: 10.3760/cma.j.cn112151-20200312-00193 32172546

[B157] Yin M. Marrone L. Peace C. G. O'Neill L. A. J . (2023). NLRP3, the inflammasome and COVID-19 infection. Qjm 116, 502–507. doi: 10.1093/qjmed/hcad011. PMID: 36661317 PMC10382191

[B158] Yiwen C. Yueyue W. Lianmei Q. Cuiming Z. Xiaoxing Y . (2021). Infection strategies of mycoplasmas: unraveling the panoply of virulence factors. Virulence 12, 788–817. doi: 10.1080/21505594.2021.1889813. PMID: 33704021 PMC7954426

[B159] Zhang L. Lai M. Ai T. Liao H. Huang Y. Zhang Y. . (2021). Analysis of mycoplasma pneumoniae infection among children with respiratory tract infections in hospital in Chengdu from 2014 to 2020. Transl. Pediatr. 10, 990–997. doi: 10.21037/tp-21-139. PMID: 34012847 PMC8107843

[B160] Zhang Y. Li J. Qiu Z. Huang L. Yang S. Li J. . (2024). Insights into the mechanism of action of pterostilbene against influenza A virus-induced acute lung injury. Phytomedicine 129, 155534. doi: 10.1016/j.phymed.2024.155534. PMID: 38583346

[B161] Zhang J. Liu J. Yuan Y. Huang F. Ma R. Luo B. . (2020). Two waves of pro-inflammatory factors are released during the influenza A virus (IAV)-driven pulmonary immunopathogenesis. PLoS Pathog. 16, e1008334. doi: 10.1371/journal.ppat.1008334. PMID: 32101596 PMC7062283

[B162] Zhang C. Meng X. Zhao H. (2022). Comparison of cell fusions induced by influenza virus and SARS-CoV-2. Int. J. Mol. Sci. 23. doi: 10.3390/ijms23137365. PMID: 35806369 PMC9266613

[B163] Zhang B. Xu S. Liu M. Wei Y. Wang Q. Shen W. . (2023). The nucleoprotein of influenza A virus inhibits the innate immune response by inducing mitophagy. Autophagy 19, 1916–1933. doi: 10.1080/15548627.2022.2162798. PMID: 36588386 PMC10283423

[B164] Zhang X. Zhang Y. Wei F. (2024). Research progress on the nonstructural protein 1 (NS1) of influenza a virus. Virulence 15, 2359470. doi: 10.1080/21505594.2024.2359470. PMID: 38918890 PMC11210920

[B165] Zhang R. Zuo Y. Li S. (2025). Mycoplasma pneumoniae MPN606 induces inflammation by activating MAPK and NF-κB signaling pathways. Microb. Pathog. 200, 107288. doi: 10.2139/ssrn.4723959 39805346

[B166] Zhao C. Pu J. (2022). Influence of host sialic acid receptors structure on the host specificity of influenza viruses. Viruses 14. doi: 10.3390/v14102141. PMID: 36298694 PMC9608321

[B167] Zheng M. Karki R. Williams E. P. Yang D. Fitzpatrick E. Vogel P. . (2021). TLR2 senses the SARS-CoV-2 envelope protein to produce inflammatory cytokines. Nat. Immunol. 22, 829–838. doi: 10.1038/s41590-021-00937-x. PMID: 33963333 PMC8882317

[B168] Zhou F. Yu T. Du R. Fan G. Liu Y. Liu Z. . (2020). Clinical course and risk factors for mortality of adult inpatients with COVID-19 in Wuhan, China: A retrospective cohort study. Lancet 395, 1054–1062. doi: 10.1016/s0140-6736(20)30566-3. PMID: 32171076 PMC7270627

[B169] Zhu M. Lu Y. Wei Y. Hong F. Ji J. Song L. (2025). The NLRP3 inflammasome activation boosts lung injury, inflammation, and macrolide resistance in mycoplasma pneumoniae pneumonia. Cytokine 195, 157014. doi: 10.1016/j.cyto.2025.157014. PMID: 40865501

[B170] Zhu Y. Sun Y. Deng X. Cao P. Li S. Yu H. . (2025). Matrix protein 1 (M1) of influenza A virus: structural and functional insights. Emerg. Microbes Infect. 14, 2558881. doi: 10.1080/22221751.2025.2558881. PMID: 40925098 PMC12451964

[B171] Zhu F. Teng Z. Zhou X. Xu R. Bing X. Shi L. . (2022). H1N1 influenza virus-infected nasal mucosal epithelial progenitor cells promote dendritic cell recruitment and maturation. Front. Immunol. 13, 879575. doi: 10.3389/fimmu.2022.879575. PMID: 35572503 PMC9095954

[B172] Ziegler C. G. K. Miao V. N. Owings A. H. Navia A. W. Tang Y. Bromley J. D. . (2021). Impaired local intrinsic immunity to SARS-CoV-2 infection in severe COVID-19. Cell. 184, 4713–4733.e22. doi: 10.1016/j.cell.2021.07.023. PMID: 34352228 PMC8299217

[B173] Zou J. Jiang M. Xiao R. Sun H. Liu H. Peacock T. . (2025). GGCX promotes Eurasian avian-like H1N1 swine influenza virus adaption to interspecies receptor binding. Nat. Commun. 16, 670. doi: 10.1038/s41467-025-55903-0. PMID: 39809757 PMC11733290

[B174] Zuo Y. Zhang R. Li S. (2024). Reviewing advancement in Mycoplasma pneumoniae P30 adhesin protein provides insights for future diagnosis and treatment. Front. Microbiol. 15, 1515291. doi: 10.3389/fmicb.2024.1515291. PMID: 39735188 PMC11671514

